# Reference Tolerance Ellipses in Bioelectrical Impedance Vector Analysis Across General, Pediatric, Pathological, and Athletic Populations: A Scoping Review

**DOI:** 10.3390/jfmk10040415

**Published:** 2025-10-22

**Authors:** Sofia Serafini, Gabriele Mascherini, Raquel Vaquero-Cristóbal, Francisco Esparza-Ros, Francesco Campa, Pascal Izzicupo

**Affiliations:** 1 Department of Medicine and Aging Sciences, University “G. D’Annunzio” of Chieti-Pescara, 66100 Chieti, Italy; sofia.serafini@phd.unich.it (S.S.); pascal.izzicupo@unich.it (P.I.); 2 Department of Experimental and Clinical Medicine, University of Florence, 50134 Florence, Italy; gabriele.mascherini@unifi.it; 3 Department of Physical Activity and Sport Sciences, Faculty of Sport Sciences, University of Murcia, 30720 San Javier, Spain; raquel.vaquero@um.es; 4 Cátedra Internacional de Cineantropometría, UCAM Universidad Católica San Antonio de Murcia, 30170 Murcia, Spain; fesparza@ucam.edu; 5 Department of Biomedical Sciences, University of Padua, 35131 Padua, Italy

**Keywords:** bioelectrical impedance vector analysis, BIVA, tolerance ellipses, R-Xc graph, body composition, reference values

## Abstract

**Background**: Bioelectrical Impedance Vector Analysis (BIVA) is a qualitative method that standardizes resistance and reactance relative to stature (R/H and Xc/H) and plots them as vectors on an R-Xc graph. This equation-free approach assesses body composition, allowing for the evaluation of hydration status and cellular integrity through tolerance ellipses. This study aimed to systematically map BIVA reference ellipses across general, pediatric, pathological, and athletic populations. **Methods**: A scoping review was conducted according to PRISMA-ScR guidelines. Five databases were searched. Extracted data included (a) sample characteristics (sample size, age, sex, BMI, country, ethnicity), (b) population type, (c) analyzer specifications, and (d) R/H and Xc/H means, standard deviations, and correlation values. **Results**: A total of 53 studies published between 1994 and July 2025 were included. From these, 508 tolerance ellipses were identified: 281 for the general population (18–92 years), 133 for children/adolescents (0–18 years), 49 for athletes, and 45 for pathological groups. Studies were primarily conducted in Europe and the Americas, using 11 analyzers with variations in measurement protocols, including body side, posture, and electrode placement. **Conclusions**: This scoping review categorizes the existing BIVA tolerance ellipses by population type, sex, age, BMI, device used, and measurement protocol. The structured presentation is intended to guide researchers, clinicians, nutritionists, and sports professionals in selecting appropriate reference ellipses tailored to specific populations and contexts.

## 1. Introduction

Bioelectrical impedance analysis (BIA) is a widely utilized technique for body composition assessment, valued for its simplicity, portability, and cost-effectiveness [1]. This method is based on the differential electrical properties of biological tissues, particularly impedance (Z) [2]. Initially, BIA was employed to examine the correlation between Z and blood flow conductive volume, later extending to total body water (TBW) [3]. By 1980, it was used to quantify distinct body composition compartments based on the electrical properties of hydrous and anhydrous tissues, as well as their respective electrolyte content [4]. From a geometrical perspective, Z is the vectorial sum of resistance (R) and reactance (Xc), expressed as Z = (R^2^ + Xc^2^)^0.5^; R reflects the opposition of biological conductors to an alternating current, primarily due to intracellular and extracellular fluids; Xc, on the other hand, represents the capacitive component of Z, arising from cell membranes, with variations influenced by membrane integrity [2]. The phase angle (PhA) is derived from the geometric relationship between R and Xc, calculated as arctangent (Xc/R) × 180°/π. It is considered a key indicator of cellular health, where higher PhA values are associated with greater cellularity, improved cell membrane integrity, and enhanced cellular function [5,6,7,8,9,10]. BIA estimates impedance by applying an alternating electrical current of low intensity through the body. In conventional BIA, impedance and its components are incorporated into single or multiple regression equations to estimate body compartments, such as fat mass (FM) and fat-free mass (FFM) [11]. However, the predictive accuracy of these equations is contingent upon the degree to which an individual’s characteristics align with those of the reference population for which the equations were originally developed [11,12,13,14,15,16]. Early predictive models were derived from heterogeneous population samples, leading subsequent research to focus on developing population-specific equations to enhance the precision of body composition estimates by bioelectrical data and/or anthropometric values [17,18,19]. Nevertheless, certain BIA devices do not allow for the selection of specific predictive equations, leading to estimations that do not account for individual variability [1,19,20]. Although substantial progress has been made in the development of population-specific predictive models, it became evident that the establishment of a comprehensive set of equations for different body compartments would require years of research and the use of gold-standard methods for validation. Moreover, even the most optimized predictive equations exhibit an inherent standard error of estimation, and the proliferation of regression models further complicates the comparability of results across different studies and populations [20]. These limitations, among others, can be partially mitigated through the bioelectrical impedance vector analysis (BIVA) method introduced by Piccoli and colleagues, which offers a standardized, qualitative approach for interpreting R and Xc relative to an individual’s stature (R/H; Xc/H, respectively) [10]. However, comparability across studies and populations, and accounting for individual variability remain important considerations [21]. In this method, R/H and Xc/H are represented as a single vector on the R-Xc graph, allowing for the assessment of soft tissues and body fluid distribution [21]. The length of the vector is inversely correlated with TBW, whereas its direction, determined by the PhA and Xc, was initially associated with body cell mass content [21]. More recently, it has been recognized as an indicator of fluid distribution between the intra- and extracellular compartments [22,23].

Unlike traditional predictive equations, this qualitative approach mitigates several methodological limitations, including regression estimation errors, technical variability in reference measurements, constraints imposed by the bioelectrical volume model (i.e., cylindrical length assumptions), and biological variability [24]. In this approach, Piccoli and colleagues introduced graphical elliptical regions, known as tolerance ellipses (50%, 75%, and 95%), to facilitate the comparison of individual vectors with normative reference values derived from a healthy general population [21]. Using the center of the ellipses as a reference, along the major axis (vertical), if the vector extends beyond the upper region of the 50% tolerance ellipse, the individual is considered to have a lower body fluid content compared to the reference population. If the vector extends beyond the 75% and 95% tolerance ellipses, it indicates that the individual has a markedly lower body water content compared to the reference population. This condition may reflect an extreme state of dehydration. However, such a deviation does not necessarily imply pathology, as individual variability in body water content exists and may differ from the average without clinical significance. Conversely, if the vector is shortened along the major axis below the 75% tolerance ellipse in the lower region, the individual is interpreted as having higher body fluids compared to the reference and potentially being in a state of hyperhydration. Regarding the minor axis (horizontal), when the vector extends beyond the left region of the 50% tolerance ellipse, it indicates a higher intracellular-to-extracellular water ratio (ICW:ECW) and body cellular mass (BCM), whereas displacement toward the right region suggests a lower ICW:ECW and BCM. Over the years, BIVA has gained popularity as a method for classifying individuals’ body composition in relation to a reference population. By considering the center of the ellipses as the mean bivariate value for the bioelectrical properties of a given population, shorter vectors indicate individuals with higher fluid content, as observed in cases of obesity or inflammatory conditions, whereas longer vectors correspond to individuals with lower total body water, such as lean individuals or those experiencing dehydration. Additionally, vectors positioned on the left side of the ellipses are generally associated with individuals possessing greater muscle mass, while rightward-shifted vectors are commonly observed in sarcopenic individuals [21]. Buffa and colleagues refined Piccoli’s BIVA (classical BIVA) approach by introducing a modified method known as specific BIVA [25].

This approach involves the simultaneous adjustment of R and Xc for both stature and the cross-sectional areas of the arm, waist, and calf. While classical BIVA was primarily designed to assess TBW, specific BIVA was developed to estimate %FM. In this modified approach, changes in vector length correspond to variations in %FM, whereas lateral displacement of the vector continues to reflect the bioelectrical PhA, as in classical BIVA [25]. Given these interpretations, the composition of the reference population remains a critical factor influencing the accuracy and applicability of both methods. Over the years, studies have validated new tolerance ellipses for various populations, including pediatric, elderly, sport-specific, and pathological groups [26,27,28,29]. Following a preliminary search, no published or ongoing scoping or systematic reviews were identified on the development, validation, and application of classical and specific BIVA reference values for body composition assessment across different populations. Therefore, we present a scoping review aimed at systematically mapping and synthesizing the available literature to identify classical and specific BIVA reference values used for classifying body composition across different populations, including healthy individuals, clinical populations, athletes, and specific demographic groups. The findings will provide a foundation for future research aimed at refining and expanding classical and specific bioelectrical impedance vector reference values to enhance their clinical and research applications.

## 2. Materials and Methods

### 2.1. Preliminary Search

In December 2024, we conducted a preliminary search using MEDLINE (PubMed), Scopus, and Open Science Framework (OSF) databases to assess the state of the art on the topic and the Joanna Briggs Institute (JBI) database of systematic review and implementation reports to identify scoping or systematic reviews that have mapped classical and specific BIVA reference values across different populations. No studies were found. The scoping review limited the search for studies published from 1994 in English. This time frame was selected because the BIVA method was developed in 1994, ensuring that all relevant studies utilizing this approach are captured within the review [21].

### 2.2. Review Questions

The present scoping review aimed to map and synthesize the available literature on classical and specific BIVA reference values. Specifically, it addressed the following questions: Which reference ellipses are available for a given population, and how many? What methodological details are reported in the development of tolerance ellipses, and to what extent do studies differ in terms of instruments and methods? This information is essential to minimize methodological errors in the application of tolerance ellipse reference values for estimating body composition and to provide a foundation for future research aimed at refining and expanding classical and specific BIVA reference values, thereby enhancing their clinical and research utility.

### 2.3. Protocol and Checklist

This scoping review followed the framework proposed by the JBI [30]. The Preferred Reporting Items for Systematic Reviews and Meta-Analyses extension for Scoping Reviews (PRISMA-ScR) was used as a guide for reporting the full findings [31] (Supplementary Table S1). In line with the JBI framework, the inclusion criteria were based on the elements of population, concept, and context [32]. This scoping review was not registered in any protocol registry (e.g., PROSPERO, OSF), consistent with current methodological guidance indicating that protocol registration is not mandatory for this study design [32].

### 2.4. Inclusion Criteria

#### 2.4.1. Participants

All participants were included in this scoping review, as the primary objective is to provide a comprehensive summary of all tolerance ellipses described in the existing literature. This approach ensures that the review captures the widest possible range of data and applications, contributing to a broader understanding of how bioelectrical impedance vector patterns and tolerance ellipses vary across populations. There were no restrictions based on demographic factors such as age, sex, or ethnicity, nor on lifestyle or health-related factors, including physical activity levels or clinical status. By adopting this inclusive approach, the review aims to reflect the diversity of populations represented in the literature and to ensure that the findings are broadly applicable across different contexts and subgroups. This inclusivity also facilitates the identification of gaps in the literature, where specific populations or conditions may have been underrepresented in previous studies.

#### 2.4.2. Concept

This review included articles containing all the bioelectrical data necessary to reconstruct tolerance ellipses for a specific population. Essential information includes the mean and standard deviation of R/H and Xc/H, for the sample, as well as their correlation values (*r*), which are required to standardize and plot vectors on the R-Xc graph. Studies lacking these parameters were excluded. Tolerance ellipses are statistical tools used to compare an individual vector with a reference population [21]. These ellipses enable both visual and analytical comparisons by considering key bioelectrical and anthropometric parameters, placing individual measurements within the broader context of population variability in terms of water content and body cellular mass [21].

#### 2.4.3. Context

For this scoping review, all populations were included, with no restrictions based on age, sex, or ethnicity. During the data extraction and synthesis process, factors such as geographic location, socio-economic status, physical activity level, sports competition level, and the presence of pathologies in the population were considered. However, studies that generate ellipses following a treatment intervention were excluded, as such interventions could influence bioelectrical variables.

#### 2.4.4. Types of Evidence Sources

We included all quantitative study designs, such as randomized controlled trials, non-randomized controlled trials, quasi-experimental studies, pre- and post-intervention studies, prospective and retrospective cohort studies, case–control studies, and cross-sectional studies, provided they reported the essential bioelectrical data (R/H, Xc/H, and r). Studies were excluded if they were literature reviews, conference abstracts, editorial commentaries, pre-prints, letters to the editor, or case reports lacking original data. Studies generating tolerance ellipses following interventions that could systematically alter bioelectrical measures were also excluded.

### 2.5. Search Strategy

Initially, we searched MEDLINE using the medical subject headings (MeSH) to identify the descriptors and their respective entry terms for the inclusion criteria, combining them with the Boolean operators (“AND” and “OR”). We included terms related to the outcome of the instrument, the statistical approach, and the reference methods to assess body composition. We did this initial search in MEDLINE (PubMed) and Scopus databases to verify search sensitivity. We analyzed the title, abstract, and keywords of the first 100 articles of both databases to identify other terms to add to the search strategy. Then, we finalized the search strategy and applied the final version to five databases: MEDLINE/PubMed, Cochrane Library, Scopus, Web of Science, and Sport Discus. A single Boolean search was developed and adapted for use across all selected databases, considering their specific features. For PubMed, the string was to incorporate MeSH terms to ensure alignment with controlled vocabulary. For Scopus and Web of Science, the same string relied on free-text keywords, as these databases do not support MeSH terms. For Sport Discus, controlled terms from the EBSCO thesaurus were considered to enhance the comprehensiveness of the search. The search strategies for each database are available as Supplementary Information (Supplementary Table S2). Studies in languages other than English were included if an English version was available. A manual search was performed within the reference lists of the included studies to identify additional studies, and any studies meeting the predefined inclusion criteria were incorporated into the final list.

### 2.6. Source of Screening and Selection of Evidence

Two authors independently performed all steps for the selection of studies to be included in the scoping review, and a third author solved the disagreements [33]. Following the completion of the search, all retrieved records were imported into EndNote for Windows (version X9, 2018; Clarivate, PA, USA). Duplicate entries were identified and removed by comparing the following fields: (a) title, authors, and publication year, and (b) title, authors, and journal name. After the removal of duplicates, the remaining records were exported to an Excel file for Windows (version 16.75.2, Microsoft, Washington, DC, USA), and organized according to key information necessary for screening, such as authors’ names, publication year, journal title, DOI, article title, and abstract. A two-step screening process was employed to determine the studies eligible for inclusion in the scoping review. In the first step, studies were screened based on their title and abstract. In the second step, a full-text review of the studies identified in the previous step was conducted to assess whether they met all predefined inclusion criteria. Finally, before the extraction of results, the authors added the eligible records found through a manual search.

### 2.7. Extraction of Results

A data extraction form was developed using an Excel file for Windows (version 16.75.2, Microsoft, Washington, DC, USA). Data extraction for each study was conducted independently by two authors to ensure accuracy and minimize bias. In cases where information or data were missing or when additional clarifications were required, the corresponding authors of the respective studies were contacted. If no response was received before the data extraction process was finalized, or if the reporting remained incomplete, the study was excluded. Once the independent data extraction was completed, the two authors engaged in a consensus procedure to resolve any discrepancies and confirm the accuracy of the extracted data. This collaborative process ensures that the final dataset was reliable and comprehensive.

### 2.8. Presentation of Results

All the data extracted were summarized in a table presenting (a) article information: the first author and the year of publication; (b) sample information: sample size, age range, sex, body mass index range (BMI), country of origin, and ethnicity of the participants; (c) sample categorization: classification of the sample into subgroups such as children and adolescents, healthy population, pathological groups (with additional details to describe the specific pathology), and athletes (including the type of sport); (d) device information: details about the instrument used to estimate the bioelectrical values; (e) result data: the mean and standard deviation of R/H and Xc/H for the sample, as well as their *r*.

## 3. Results

### 3.1. Search

**Figure 1:**
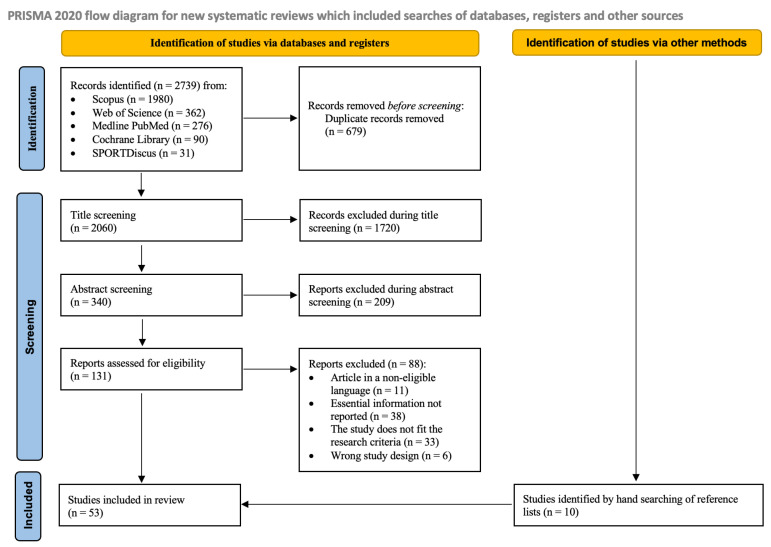
Flowchart of the study selection and inclusion process in the scoping review [31]. The figure represents the process of identification, screening, and final inclusion of 53 studies.

The initial search across the five databases yielded 2060 reports after duplicate removal. Most full-text reports were excluded due to missing values required for constructing the tolerance ellipses for the reference population (Supplementary Table S3). Additionally, manual research identified ten studies. In total, 53 studies were included (Figure 1).

### 3.2. Characteristics of Reports

**Figure 2:**
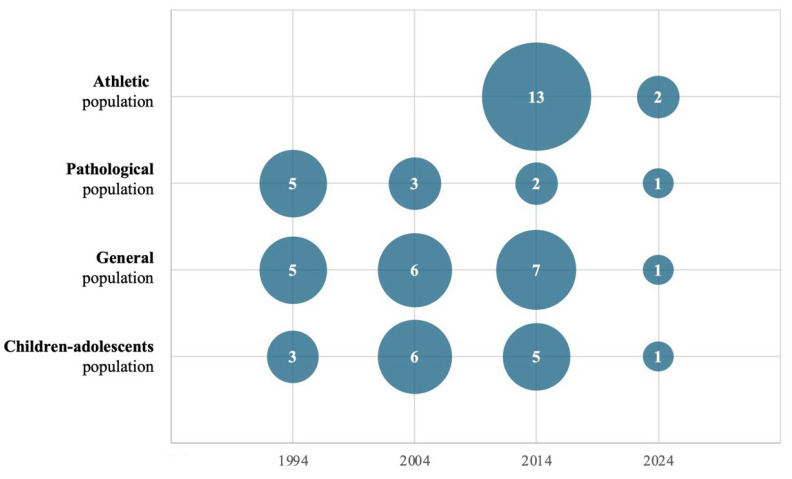
Bubble plots of the study frequency by population category and decade. Bubble plots showing the frequency of studies published by decade, categorized by population group. The y-axis represents the population categories, while the x-axis represents the decades in which the studies were published. The size of the bubbles is proportional to the number of studies published in each decade, according to the population category.

**Figure 3:**
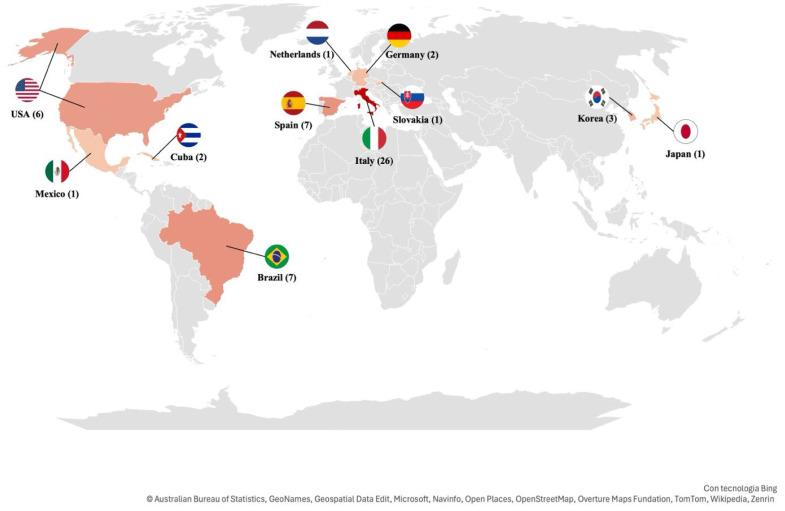
Map of countries of origin and number of studies of tolerance ellipses. The figures represent the nationality of the participants evaluated in the 53 studies included in the scoping review. Three studies were conducted in more countries: Piccoli et al. [37] in Italy and the United States; Jensen et al. [66] in Germany, Japan, and Mexico; and Ibanez et al. [65] in Italy and Spain. For this reason, the maps reported 57 studies.

The reports analyzed included a wide range of sample characteristics, with a total of 508 tolerance ellipses described and classified into four distinct groups: the general population, children and adolescents, individuals with pathological conditions, and athletes. This topic has been researched for almost 3 decades (1994–2024), and the first study identified on the topic was conducted by Piccoli and published in 1994 [21]. During the first decade (1994–2003), five articles were published on the general population [29,34,35,36,37], five on the pathological population [29,38,39,40,41], and three on children and adolescents [26,42,43]. In the second decade (2004–2013), six articles focused on children and adolescents [44,45,46,47,48,49], five on the general population [25,44,49,50,51,52], and three on the pathological category [50,52,53]. In the most recent decade (2014–2023), 13 articles were published on the athletic population [23,28,54,55,56,57,58,59,60,61,62,63,64], seven on the general population [27,65,66,67,68,69,70], five on children and adolescents [71,72,73,74,75], and two on the pathological population [68,70] (Figure 2). From 2024 to July 2025, five articles were published [76,77,78,79,80]. Tolerance ellipses were identified in 11 countries across all continents. The continent with the largest number of reports was Europe, with 37 reports [23,26,27,28,29,35,37,38,39,40,41,42,43,44,45,47,52,53,54,55,56,57,59,60,61,62,63,64,65,66,69,73,74,77,78,80], followed by America (16 reports) [25,34,36,37,46,48,49,50,51,58,66,68,71,72,75,76], and Asia (4 reports) [66,67,70,79] (Figure 3).

### 3.3. Characteristics of the Population

A total of 508 tolerance ellipses were extracted from 53 studies and classified into four distinct groups (general population, children and adolescents, pathological, and athletes) to systematically organize and effectively synthesize the findings (Supplementary Table S4).

#### 3.3.1. General Population

With 19 studies [25,27,29,34,35,36,37,44,49,50,51,52,65,66,67,68,69,70,80], the largest category consisted of 281 tolerance ellipses, with 143 tolerance ellipses for females and 138 for males, across an age range of 18 to 92 years (Supplementary Table S5). Participants exhibited diverse ethnic backgrounds, with 18 tolerance ellipses for the Asian population [66,67,70], 27 for the Caucasian [27,29,35,69,80], 4 for the Caucasian-Hispanic [36], 18 for Mexican [66], 8 for multiethnic [25,49], 37 for Mexican-American [37], 36 for Non-Hispanic Black [37], and 36 tolerance ellipses for Non-Hispanic White population [37]. Ethnicity was unspecified in 92 tolerance ellipses [44,50,51,52,65,68]. The BMI within this group ranged from 16 to 50 kg/m^2^ (Supplementary Table S5).

#### 3.3.2. Children and Adolescents

Due to the dynamic changes in body composition and bioelectrical parameters during growth, this category was further subdivided into three groups: infancy (0–3.99 years), childhood (4–11.99 years), and adolescence (12–18 years). A total of 133 tolerance ellipses were identified across 15 studies: 43 for infants (10 for females, 10 for males, and 23 for both sexes) [26,42,43,46,47,48,49,72,73,74,75,78], 39 for children (13 for females, 13 for males, and 13 for both sexes) [26,44,45,49,71,73], and 51 for adolescents (23 for females, 25 for males, and 3 for both sexes) [26,44,49,71,73]. Participants in this group ranged in age from one day after birth to 18 years. Participants exhibited diverse ethnic backgrounds, with 33 tolerance ellipses for Caucasians [26,42,43,45,78] and 24 for multiethnic populations [46,48,49,72,75], while the ethnicity of the remaining 76 ellipses was not specified [44,47,71,73,74]. Their BMI values ranged from 9 to 40 50 kg/m^2^ (Supplementary Table S6).

#### 3.3.3. Pathological Population

The third group comprised 45 tolerance ellipses representing individuals with pathological conditions (20 for females, 24 for males, and 1 for both), derived from 11 studies [29,38,39,40,41,50,52,53,68,70,79], with ages ranging from 18 years to over 80 years. Specifically, 2 tolerance ellipses described the bioelectrical values of individuals with cancer [41], 8 for those with cirrhosis [40], 1 for individuals with chronic obstructive pulmonary disease (COPD) [29], 2 for those with edema [39], 12 for populations undergoing hemodialysis [38,50,53], 3 for individuals with hip fractures [79], 2 for those with obesity [39], 9 for individuals with Parkinson’s disease [52], and 6 for those with sarcopenia [68,70]. Among these participants, 5 tolerance ellipses were for Asians [70,79], 15 for Caucasians [29,38,39,41,53], and 25 had an unspecified ethnic background [40,50,52,68]. Their BMI ranged from 19 to over 42 kg/m^2^ (Supplementary Table S7).

#### 3.3.4. Athletic Population

With 15 studies [23,28,54,55,56,57,58,59,60,61,62,63,64,76,77], the athlete group comprised 49 tolerance ellipses, with 12 for females and 37 for males, aged between 12 and 41 years. Specifically, twelve tolerance ellipses were created for soccer players [54,58,59,61,81], six for road cyclists [57], six for mixed sport [23], four for CrossFit^®^ athletes [77], four for endurance athletes [28,76], four for volleyball players [56], two for handball players [62], two for team sports [28], two for strength athletes [76], two for velocity-power athletes [28], two for bodybuilders [64], one for ultra-endurance triathletes [55], one for marathon runners [60], and one for soccer referees [63]. Among these participants, 24 tolerance ellipses were for Caucasian participants [23,54,56,60,62,64,77], 1 for multiethnic populations [63], and in 20 tolerance ellipses, the ethnicity was not specified [28,55,57,58,59,61,76]. The BMI ranged from 20 to 26 kg/m^2^ (Supplementary Table S8). For age and BMI, some studies used a sample with a limited range of age and BMI, while others incorporated a broader range.

### 3.4. Sample Size of Reports

The sample size used for the derivation of tolerance ellipses varied considerably. The study by Siváková et al. [52] had the smallest sample size, with only two participants, whereas the study by Bosy-Westphal et al. [44] had the largest, including 18,824 participants. In the general population, the majority of tolerance ellipses (n = 189; 67.03%) were generated using more than 100 participants [25,27,34,35,36,37,44,49,50,52,65,66,67,69,80]. Similarly, 83.46% (n = 111) of the tolerance ellipses for children and adolescents were generated from studies with sample sizes exceeding 100 participants [26,42,43,44,45,46,47,48,49,72,73,74,75]. Finally, within the pathological and athletic populations, 12 tolerance ellipses (26.67%) [29,38,39,40,79] and 22 tolerance ellipses (46.81%) [23,28,54,56,57,59,61,77] were derived from sample sizes greater than 100 participants, respectively.

### 3.5. Tolerance Ellipse Method

Among the 53 papers, 46 reported values for classical BIVA [26,28,29,34,35,36,37,38,39,40,41,42,43,44,45,46,47,48,49,50,51,52,53,54,55,56,57,58,60,61,62,63,64,66,67,69,70,71,72,73,74,75,76,78,79,80], 6 provided data for both classical and specific BIVA [23,25,27,59,68,77], and 1 study reported values exclusively for specific BIVA [65]. A total of 508 tolerance ellipses were extrapolated, of which 16 were identified for specific BIVA [23,25,27,59,65,68,77]. Specifically, nine of these ellipses were for the general population [25,27,65,68], two for the pathological populations [68], and five for athletes [23,59,77]. For the classical BIVA, 272 tolerance ellipses were for the general population [29,34,35,36,37,38,39,40,41,44,49,50,51,52,53,66,67,69,70,79,80], 133 for the children-adolescent population [26,42,43,44,45,46,47,48,49,67,71,72,73,74,75,78], 43 for the pathological population, and 44 for athletes [23,28,54,55,56,57,58,59,60,61,62,63,64,76,77].

### 3.6. Statistical Analysis

The linear correlation coefficient (*r*) between R/H and Xc/H varied across the analyzed populations, influencing the shape and orientation of the tolerance ellipses. Only one study reported a negative correlation in five tolerance ellipses for children aged from 1 day to 12 months [43]. The majority of the tolerance ellipses (48%) had a correlation ranging from 0.40 to 0.69, while 39.76% had a correlation between 0.70 and 0.89. In the general population, only one ellipse had a correlation lower than 0.10 [68], while the others had r values ranging from 0.41 to 0.88. In the children and adolescents’ category, 8 ellipses had a correlation below 0.10 [43,47], while the others ranged from 0.10 to 0.91. In the pathological group, the correlation ranged from 0.14 to 1.00, while in athletes, it ranged from 0.39 to 0.81.

### 3.7. Analyzer

**Figure 4:**
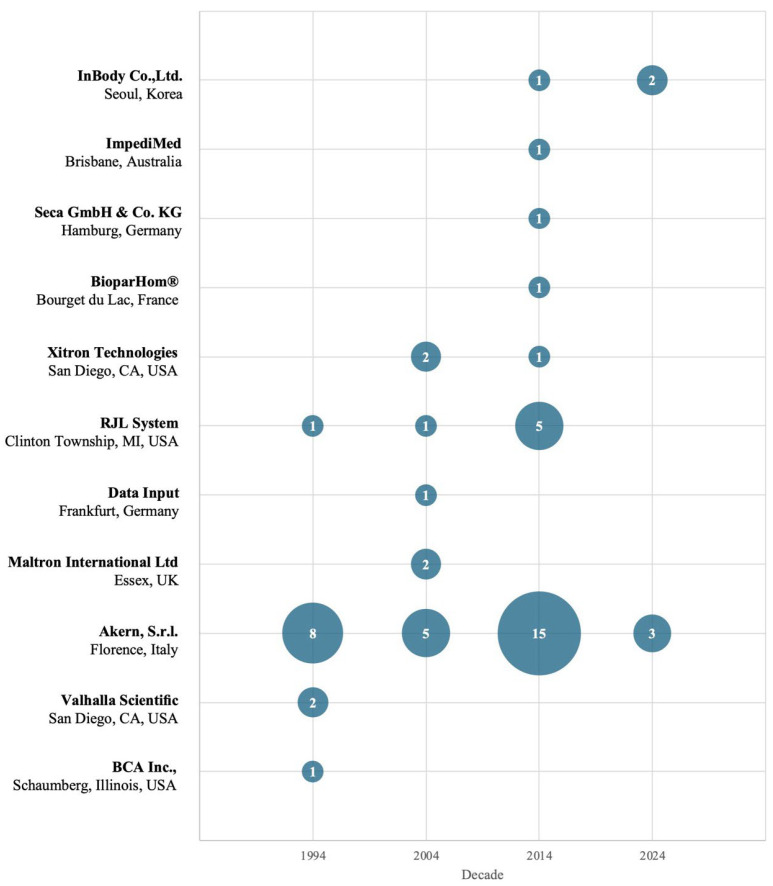
Bubble plots showing the frequency of analyzer manufacturing companies by decade. The y-axis represents the company of the analyzer model used to collect bioelectrical data. The x-axis represents the decades in which the studies were published. The size of the bubble is proportional to the number of studies published in the decade.

Analyzers from different companies were employed in the study protocols to collect bioelectrical data (Figure 4). During the first decade (1994–2003), six different analyzer models from three companies were used. The most frequently utilized were the BIA 101, BIA-109, and STA/BIA models from Akern Srl (Florence, Italy) reported in eight studies [26,35,36,38,39,40,41,42,43]. Additionally, two studies used the 1990B analyzer from Valhalla Scientific (San Diego, CA, USA) [34,37], while one study employed the BCA analyzer from BCA Inc. (Schaumberg, IL, USA) [36] and one study the Quantum Analyzer from RJL System, Inc. (Clinton Township, MI, USA) [29]. In the second decade (2004–2013), a greater variety of analyzers was introduced. Five studies used the BIA-101 analyzer from Akern Srl (Florence, Italy) [45,47,51,52,53], while two studies utilized the Xitron Hydra ECF/ICF Bio-Impedance Analyzer System Model 4200 from Xitron Technologies (San Diego, CA, USA) [25,48]. Additionally, one study employed the Quantum II analyzer from RJL System, Inc. (Clinton Township, MI, USA) [46], and two studies used the Bioscan BL-960141 from Maltron International Ltd. (Essex, UK) [49,50]. Last, Bosy-Westphal et al. utilized BIA 2000-S from Data Input (Frankfurt, Germany) [44]. In the last decade (2014–2024), Akern analyzers (Florence, Italy) became the most used. Of the 15 studies, 9 utilized the BIA 101 Anniversary model [23,27,28,56,59,60,61,62,63], 4 studies used the BIA-101 [54,57,65,74], 1 study employed the BIA 101 and the BIA 101 BIVA PRO [69], and 1 study used the BIA 101 BIVA PRO [64]. Other analyzers included five studies using models from RJL System, Inc. (Clinton Township, MI, USA) (BIA 103-A; Quantum II; RJL 101-A; Quantum Desktop RJL-101; Model 101 Quantum, respectively) [58,70,71,72,73]. In the last five studies, one each used one of these models: the Xitron Hydra ECF/ICF Bio-Impedance Analyzer System Model 4200 (Xitron Technologies, San Diego, CA, USA) [75], InBody S10^®^ (InBody Co., Ltd., Seoul, Republic of Korea) [67], Z-Métrix^®^ (BioparHom^®^, Bourget du Lac, France) [55], Seca mBCA 514 (Seca GmbH & Co. KG, Hamburg, Germany) [66], and BIA Imp DF50 Body Composition Analyzer (ImpediMed, Brisbane, Queensland, Australia) [68]. From 2024 to the present, three studies employed Akern models (Florence, Italy; BIA-101, BIA 101 Anniversary and BIA 101 BIVA PRO) [77,78,80], and one study employed the InBody 770 analyzer [76], while another utilized the InBody S10^®^ [79]; both instruments were manufactured by InBody Co., Ltd. (Seoul, Republic of Korea).

### 3.8. Body Side

Different analyzer configurations were used to assess bioelectrical data (Supplementary Table S4). Among the 53 studies, 48 utilized bioimpedance analyzers with a tetrapolar configuration, employing 4 electrodes in a hand–foot system [23,25,26,27,28,29,34,35,36,37,38,39,40,41,42,43,44,45,46,47,48,49,50,51,52,53,54,56,57,58,59,60,61,62,63,64,65,68,69,70,71,72,73,74,75,77,78,80]. Participants were required to lie in a supine position during the measurement. The sensor electrodes were placed on the dorsal surface of the wrist (between the ulna and radius) and on the anterior surface of the ankle (between the prominent bone structures). The injector electrodes were positioned on the dorsal surface of the third proximal phalanx of both the hand and foot. A minimum distance of 5–6 cm was maintained between the sensor and injector electrodes to ensure accurate measurements. Two studies employed the same analyzer with a tetrapolar eight-point tactile electrode system, with participants positioned in the supine position [67,79]. One study did not specify the participants’ position; however, the analyzer used in this study was adaptable for supine, sitting, or orthostatic positions, and two electrodes were placed on the right wrist and two on the right ankle, with at least 5 cm spacing [55]. Finally, two studies conducted bioimpedance analysis in an orthostatic position [66,76]. In the first study, the Seca mBCA 514 (Seca GmbH & Co. KG, Hamburg, Germany) was used, which featured six electrodes on each side of the ascending handrail, with two selected based on participant stature [66]. This required an upright stance with outstretched arms. Additionally, two electrode pairs were positioned on the feet, enabling segmental impedance measurement with an eight-electrode technique. In the second study, the InBody 770 (InBody Co., Ltd., Seoul, Republic of Korea) was used at a single frequency of 50 kHz, where participants positioned their heels on round silver electrodes, placed their feet on foot electrodes, put their thumbs on thumb electrodes, wrapped their fingers around bottom electrodes, and kept their arms slightly extended (~15°) from the torso [76].

### 3.9. Measurement Error

Most studies reported performing daily or weekly calibration of the analyzer before data collection using known impedance circuits. They also provided accuracy or error values to indicate technical reliability. Additionally, some studies assessed repeatability and intra/inter-individual variability, reporting coefficients of variation for within-day, between-day, and inter-observer measurements. Finally, a few studies examined the center effect, which represents the combined variability introduced by both the operator and the instrument (Supplementary Table S4).

## 4. Discussion

This scoping review aimed to provide a comprehensive synthesis of the existing literature on classical and specific BIVA reference tolerance ellipses for body composition assessment. By systematically mapping studies that have developed, validated, and applied these reference values across various populations, our findings provide scholars and practitioners with a valid benchmarking tool for BIVA, as well as valuable insights into the strengths and limitations of current BIVA methodologies.

### 4.1. Characteristics of the Population and Sample Size

A total of 53 papers yielded 508 tolerance ellipses. The general population, with 281 ellipses, represented the largest percentage (55.14%) of tolerance ellipses, followed by the children–adolescent population with 133 ellipses (26.28%), athletes (49 ellipses; 9.68%), and the pathological population (45 ellipses; 8.89%). The observed differences in the number of ellipses among groups likely reflect the broader availability of data on apparently healthy individuals, who are more commonly represented in the literature compared to more specific or clinical populations.

#### 4.1.1. General Population

This category encompasses a wide age range (18 to 92 years), including subgroups such as young adults, adults, older adults, and the elderly, as well as a large range of BMI values (16 to 50 kg/m^2^), including overweight and obese individuals. This wide range enabled the creation of tolerance ellipses stratified by sex, age, BMI, and ethnicity. Piccoli et al. stratified the population into six age categories (20–29 years, 30–39 years, 40–49 years, 50–59 years, 60–69 years), three BMI categories (19–25, 25–30, and 30–35), and three ethnicity categories (Mexican-American, Non-Hispanic Black, and Non-Hispanic White), with a minimum of 29 participants per subgroup [37]. Similarly, Jensen B. et al. created tolerance ellipses stratified by sex, age, and BMI for Asian, Mexican, and Caucasian ethnicities [66]. Bosy-Westphal et al. stratified for sex, age (18–19 years, 20–29 years, 30–39 years, 40–49 years, 50–59 years, 60–69 years, and > 70 years) and BMI (18.5–25, 25–30, 30–35, 35–40, and 40–50). Although the authors reported that the participants were German, no details were provided about their ethnicity [44]. Nescolarde et al. stratified a multiethnic Cuban population for sex and age, adopting a wider range (17–59 years and 60–80 years) [49]. Campa et al. created tolerance ellipses for the Italian adult population with an age range from 18 to 65 years [69]. Finally, Siváková et al. stratified the Slovakia population for sex and age (18–28 years, 29–39 years, 40–49 years, 50–59 years, 60–69 years, 70–79 years, 80–92 years) but did not provide information regarding BMI and ethnicity [52].

**Figure 5:**
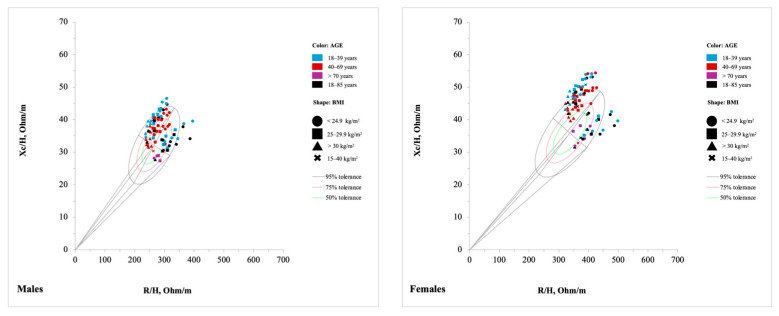
General population. Mean vector data from literature plotted on the new reference ellipses for males and females [69].

When the bioelectrical impedance vectors derived from these reference populations, stratified by sex, age group, and BMI, were plotted against the newly developed tolerance ellipses for healthy Italian adults aged 18 to 65 years [69], most individuals were positioned in the upper-left quadrant of the R-Xc graph and were predominantly located within the 95% tolerance ellipse, with the greatest concentration in the 50% and 75% percentiles (Figure 5). This vector positioning is classically associated with favorable bioelectrical properties, including a higher ICW:ECW and greater TBW, characteristics reflective of healthy tissue composition and hydration status. However, as highlighted by Campa and colleagues, the physical characteristics and body composition of populations have changed over the past thirty years, leading to a shortening of the vector and its leftward shift. The findings of this scoping review are largely consistent with this interpretation [69]. Vector characteristics were primarily influenced by age and BMI. Younger individuals exhibited a tendency for longer vectors compared to older participants, suggesting higher cellular mass and membrane integrity, along with greater intracellular water content [37,52,66,67]. As BMI increased, vector length tended to decrease, with a downward shift in orientation, consistent with alterations in fluid distribution and reduced cellular mass observed in overweight and obese individuals. Aging was associated with a gradual rightward shift of the vectors: adults aged up to 69 years remained predominantly within the left portion of the ellipses [34,35,37,49,52,66,67,68], while individuals over 70 years, particularly those over 85, were more frequently located on the right side [27,36,52,68,70,80]. This trend reflects well-established physiological changes such as sarcopenia and reduced hydration status associated with advanced age. Differences observed between the newly developed ellipses [69] and those derived from previous reference populations, as well as among various populations, must be interpreted with caution. First, Campa et al. used bioimpedance devices from Akern, which may introduce measurement variability when compared to reference populations assessed using instruments from other manufacturers [69]. Moreover, shifts in body composition trends over the past three decades, along with differences in measurement protocols (e.g., electrode placement, posture during assessment), may contribute to deviations in vector positioning. Ethnic variability also plays a key role in bioelectrical parameters, as previously reported by Piccoli et al., further complicating direct comparisons between populations [37]. Thus, such comparisons should be considered informative rather than prescriptive, acknowledging methodological and demographic differences that may influence reference values.

#### 4.1.2. Children–Adolescent Population

The children and adolescent categories comprise distinct developmental stages (infancy, childhood, and adolescence), each characterized by rapid and heterogeneous changes in body proportions, size, and composition from birth to complete maturation. Consequently, authors typically employed narrower age intervals compared to those used for adult populations. De Palo et al. stratified the Italian Caucasian pediatric population into finely segmented age groups (2–3, 4–5, 6–7, 8, 9, 10–11, 12, 13, 14–15 years) [26]. For children aged 2 to 13 years, sex stratification was not applied, as the 95% confidence ellipses of the mean impedance vectors overlapped between boys and girls within each age group. However, in the 14–15-year-old subgroup, statistically significant sex-based differences in vector positioning were observed, justifying the introduction of sex-specific ellipses. A similar methodological approach was adopted by Nescolarde et al., who stratified the Cuban pediatric population into the following age groups: 2–3, 4–5, 6–7, 8–9, 10–11, 12, and 13–16 years. Sex stratification was only applied from 13 years of age onward, as no significant differences were detected in children aged 2 to 12 years [49]. Bosy-Westphal et al. stratified German participants by sex and age groups with three-year intervals (6–9, 10–13, 14–17 years), and by BMI (9–13, 13–15, 15–17, 17–25, 25–30, 30–35 kg/m^2^), thereby including normal-weight, overweight, and obese categories [44]. Several studies focused on infancy, analyzing age groups ranging from a few days to three months [42,43,46,47,48,72,74,82]. Guida et al. stratified a cohort of Italian 8-year-old children by BMI category: normal weight, overweight, and obese [45]. Other studies focused on single age ranges and diverse nationalities; some were multiethnic [46,48,49,72,75], although the majority were conducted in Caucasian populations [26,42,43,45,78]. Compared to adults, many ethnic and age-specific pediatric subgroups remain underrepresented in the current literature.

**Figure 6:**
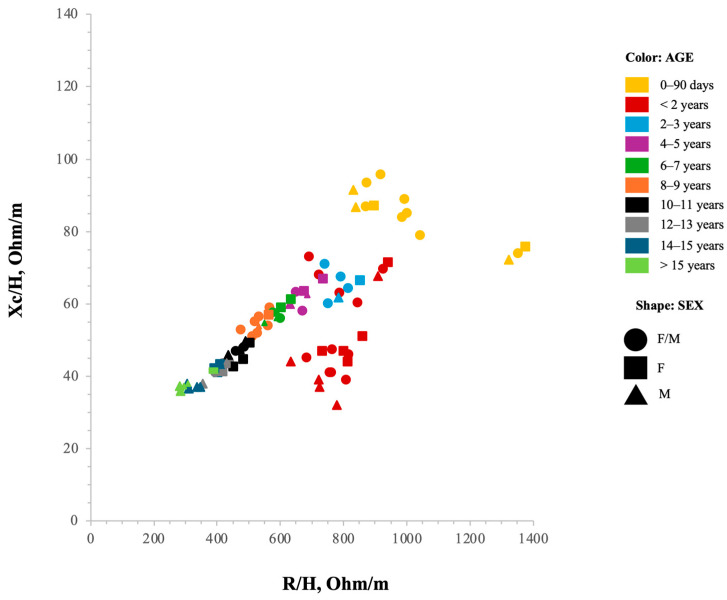
Children and adolescents’ mean vector data from the literature.

The distribution of bioelectrical vectors in children and adolescents, stratified by sex and age group, is presented in Figure 6. Overall, the patterns were consistent across subgroups, reflecting physiologically coherent trends in body composition during growth. As age increases, vector length gradually shortens, likely indicating the progressive accumulation of cellular mass during development. Sex-based differences become more evident after the age of 13, corresponding with the biological onset of puberty [26,71,73]. These differences are reflected in a shorter impedance vector in males, positioned lower and to the left on the R-Xc graph, and are indicative of differences in cellular mass and TBW. These findings align with the expected timeline of growth and maturation, with peak pubertal growth occurring, on average, at 11.7 years in girls and 13.4 years in boys, followed by a subsequent deceleration in growth rate. Importantly, because bioelectrical parameters are normalized for stature, the observed changes in vector position cannot be attributed solely to linear growth [83]. Rather, they reflect underlying alterations in body composition associated with biological maturation [84]. This interpretation is supported by findings from Buffa, Floris, and Marini, who documented significant trends toward vector shortening and changes in vector orientation in a cohort of 143 Sardinian girls before and after menarche, consistent with pubertal progression [85]. As in adults, observed differences between studies may be reasonably attributed to variation in age groupings, sex ratios, ethnicity, and technical aspects such as the type of device used, skin-electrode distance, and placement protocols.

#### 4.1.3. Pathological Population

This category includes 45 tolerance ellipses, covering individuals aged between 18 and over 80 years, with BMI values ranging from 19 to more than 42 kg/m^2^. Only two studies stratified participants by sex and age [50,52]. In general, pathological populations in the considered studies were represented by smaller samples compared to the general population. Consequently, most authors generated tolerance ellipses based on the entire sample, without applying stratification. On the other hand, when stratification was applied, some subgroups became so small that they were no longer representative. For instance, in the study by Siváková et al. involving participants with Parkinson’s disease, each sex- and age-stratified subgroup included fewer than 12 individuals [52]. Among the 45 ellipses, 24 were from subjects with metabolic diseases (hemodialysis, cirrhosis, and obesity) [38,39,40,50,53], 9 from neurological diseases (Parkinson’s disease) [52], 9 from skeletal-muscle diseases (sarcopenia and hip fractures) [68,70,79], 2 from subjects with cancer [41], and 1 from COPD [29]. An aspect that is worth consideration when referring to reference bioelectrical values relative to the pathological population is that most of the ellipses were derived from small samples and specific geographical areas (Supplementary Table S7). Thus, caution must be taken in interpreting comparisons, especially considering the wide range of diseases that significantly impact fluid dynamics and body composition [86]. While BIA has already been investigated in several pathological conditions, most diseases lack tolerance ellipses, though fluid distribution plays a crucial role in the disease process. For example, studies on pathologies such as heart failure, hypertension, lymphedema, and diabetes highlight the diagnostic usefulness of BIVA [82,87,88,89]. Thus, expanding the availability and use of specific tolerance ellipses to better capture the complexities of fluid dynamics in various pathological states could lead to more targeted clinical interventions and management strategies.

**Figure 7:**
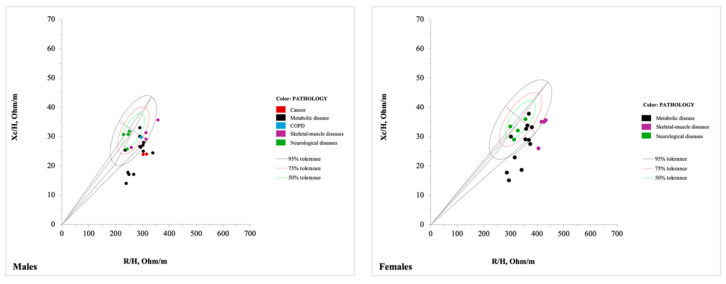
Pathological population. Mean vector data from literature plotted on the new reference ellipses for males and females [69].

The data of pathological populations plotted against the newly developed tolerance ellipses for adult individuals aged 18 to 65 years [69] can be seen in Figure 7. Different diseases were characterized by distinct bioelectrical vector patterns, with similar trends in males and females. In individuals with metabolic diseases, the vector tends to be shorter and shifted further to the right [38,39,40,50,53,70,79] while in oncological patients, the displacement of the vector was primarily characterized by a reduction in the Xc component [41], resulting in a lower PhA. It has been suggested that this impedance pattern reflects altered electrical properties of body tissues, particularly in relation to body cell mass, as the Xc component reflects the capacitive properties of cell membranes in soft tissues. These distinct BIVA patterns may be indicative of overall tissue changes, involving both mass and structural alterations. Importantly, a change in total body hydration can likely be excluded in oncological patients, given that the R component, strongly associated with TBW, remained similar to that of healthy subjects. The neurological disease patients were positioned more to the left with respect to other conditions, probably because none of the patients had a BMI below 19, and mean BMI values ranged from overweight to obese, reflecting the absence of severe malnutrition or dehydration in the sample (Siváková et al. [52]).

#### 4.1.4. Athletic Population

Among athletes, only a limited number of sport categories were represented by specific tolerance ellipses, and some sports with specific physiological characteristics are underrepresented. The most frequently included disciplines were team sports (e.g., soccer, volleyball, handball), cycling (specifically all-rounders and sprinters), and velocity/power sports such as CrossFit^®^ and bodybuilding. Three studies developed tolerance ellipses by grouping multiple disciplines rather than focusing on a single sport [23,28,76]. Campa et al., for example, produced sex-stratified ellipses for three broad categories: endurance sports (e.g., cycling, marathon, pentathlon, cross-country skiing, rowing, and triathlon), velocity/power sports (including jumping, throwing, short-distance running, badminton, boxing, CrossFit^®^, judo, karate, kickboxing, rhythmic gymnastics, short-distance swimming, and tennis), and team sports (e.g., basketball, field hockey, handball, rugby, soccer, volleyball, and water polo) [28]. Similarly, Marini et al. generated ellipses for male and female athletes across several sports, including athletics, basketball, handball, judo, karate, pentathlon, rugby, soccer, swimming, triathlon, and volleyball [23]. Abdelnour et al. on the other hand, distinguished between strength and endurance athletes through two sex-stratified ellipses, yet they did not report the specific sports included [76]. A notable gap emerged for weight-class and esthetic sports such as boxing, weightlifting, powerlifting, wrestling, judo, dance, and gymnastics, where athletes often engage in practices such as weight-cutting, which consists of rapid weight loss, primarily through fluid depletion over just a few days or hours [90]. Importantly, BIVA has proven effective in detecting fluid loss and subsequent rehydration following an acute training session [91,92].

Unlike in studies on adults, tolerance ellipses among athletes were not stratified by age or BMI. This may be expected, as athletes within the same sport generally share similar body composition profiles. Furthermore, sports inherently classify athletes by age and sex, resulting in relatively homogeneous groups. Finally, some studies may have lacked sufficient sample sizes to support the generation of separate ellipses. Of the 47 tolerance ellipses identified, only 11 were developed specifically for female athletes, revealing a substantial gap in the literature. This underrepresentation persists despite gender equity in sports is growing. While this disparity may rooted in historical, structural, and sociocultural factors, many disciplines, particularly those with a strong tradition of professional male leagues (e.g., soccer, cycling, weightlifting), still provide fewer competitive opportunities for women, resulting in smaller sample sizes in research studies on high-performance athletes [93,94]. Additionally, the greater physiological and bioelectrical variability observed in women compared to men, linked to hormonal fluctuations and the use of contraceptives, makes data collection more complex [81,95,96]. In the first two decades (1994–2004), there were no publications on athletes, suggesting that the use of bioimpedance was initially focused on other populations, particularly those with medical conditions (Figure 2). This may be because, initially, Piccoli et al. developed this method for the clinical monitoring of patients with body composition and fluids-related issues, such as those suffering from metabolic or renal diseases [21]. Since 2014, there has been an increase in publications focusing on athletes [23,28,54,55,56,57,58,59,60,61,62,63,64], reflecting a growing recognition of athletes as a distinct population with bioelectrical characteristics that differ from those of the general or healthy population.

**Figure 8:**
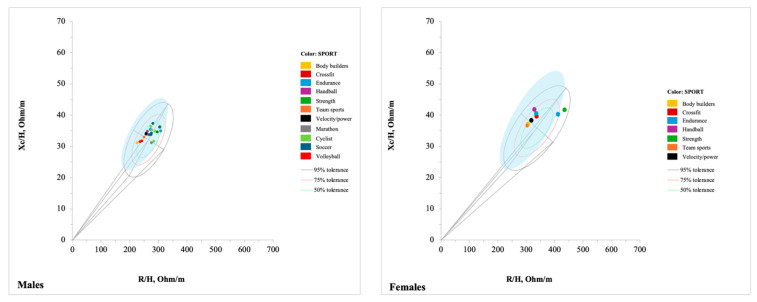
Athletic population. Mean vector data from literature plotted on the new reference ellipses for the healthy population [69] and male and female athletes (tolerance ellipse in light-blue) [28].

After plotting athletes in the R-Xc mean graph (Figure 8), it can be observed that they tend to cluster more closely than other populations. This is likely due to their narrower age range and shared characteristics, such as good cellular health and favorable body composition. Athletes practicing resistance or power-oriented disciplines such as bodybuilding and volleyball locates in the lower-left quadrant of the R-Xc graph reflecting substantial muscle mass [56,64]. On the other hand, volleyball athletes, who typically have taller stature, may display shorter vectors as a result of the standardization of bioelectrical values by stature. Endurance disciplines were located mainly on the top and right side [28,76]. This pattern corresponds to a lower absolute lean mass, reduced total body water, and an overall slimmer body composition, consistent with sport-specific physiological adaptations aimed at optimizing aerobic efficiency, energy expenditure, and thermoregulation. These athletes maintain vector positions within the athletic tolerance ellipse (light-blue, Figure 8) yet diverge significantly from those practicing strength- or power-dominant sports, underlining the impact of sport-specific training on body composition and cellular properties as captured by vectorial bioimpedance [28].

### 4.2. Tolerance Ellipse Method

A total of 508 tolerance ellipses were identified, with 16 specifically corresponding to the specific BIVA, highlighting the predominant use of classical BIVA. The specific BIVA, introduced in 2013, represents a modification of the classical approach aimed at more accurately assessing the relative proportion of fat mass [25]. This modification is based on Ohm’s law, which suggests that impedance is influenced by both an individual’s stature and body cross-sectional area. Unlike classical BIVA, which primarily uses bioelectrical impedance data and stature, specific BIVA also requires measurements of arm and calf circumferences for both limbs, improving the precision of fat mass estimation. Given that specific BIVA was developed 20 years after classical BIVA, it is expected that fewer tolerance ellipses would be available for this method. Additionally, the inclusion of extra anthropometric variables increases the time and complexity of data collection, making its application more challenging in populations such as those with pathological conditions or children. Nevertheless, specific BIVA should be more widely utilized in both medical and sports fields, as it has demonstrated accuracy, particularly in evaluating FM, and provides an additional tool for assessing body composition across all population categories [25].

### 4.3. Statistical Analysis

The correlation coefficient between R/H and Xc/H plays a role in the shaping of the tolerance ellipse. A stronger positive correlation results in more elongated ellipses oriented diagonally, while a weaker correlation or negative correlation leads to more circular or differently oriented ellipses [21]. Across the analyzed studies, most tolerance ellipses (48%) exhibited a moderate correlation (*r* = 0.40–0.69), and 39.76% showed a strong correlation (*r* = 0.70–0.89). This suggests that, in most cases, R/H and Xc/H have a consistent linear relationship, influencing the orientation and elongation of the ellipses. However, variability was observed across different population groups, highlighting the influence of physiological characteristics on bioelectrical impedance parameters.

### 4.4. Analyzer and Body Side

A detailed description of the type of analyzer used and the procedure followed for the assessment is crucial to ensure methodological consistency and reproducibility. The Akern Srl and RJL Systems are widely used across various countries for collecting bioelectrical data and generating tolerance ellipses. When assessing body composition, it is essential to account for the specific bioelectrical impedance analyzer employed, as significant variations in bioelectrical values can occur depending on the instrument. Graves et al. reported notable differences in resistance values (±36 Ohms) between two different analyzers, highlighting the potential impact of device selection [97]. These discrepancies are particularly relevant when comparing a sample to a reference population. To minimize measurement errors, it is therefore essential to use reference population tolerance ellipses derived from the same type of analyzer. Similarly, the polar configuration and electrode placement are crucial factors in minimizing measurement errors. Most studies used a tetrapolar configuration with four electrodes in a hand–foot system, maintaining 5–6 cm between them. However, two studies employed analyzers with an eight-point tactile electrode system in the supine position [67,79]. A recent study compared raw measures from three BIA devices (tetrapolar supine, octopolar supine, and octopolar standing devices) to assess inter-device variation. The results revealed substantial differences in R (up to 24.6%) and Xc (up to 43.7%) between devices, even under standardized testing conditions. These discrepancies led to divergent body composition estimates and BIVA tolerance ellipses with no overlap across systems, highlighting the limited comparability of results derived from different technologies. Furthermore, two other studies conducted measurements in an orthostatic position [66,76]. These methodological variations are relevant, as body position during measurement influences bioelectrical parameters. The orthostatic position may yield different values compared to the supine position due to fluid redistribution in the body [2]. Differences in measurement instruments and protocols can impact the comparability of results with the selected tolerance ellipses. This is particularly important in clinical and sports settings, where measurement accuracy is critical for diagnostic assessments and body composition monitoring. Increasing the number of tolerance ellipses specific to different analyzers should be considered to reduce errors and enhance methodological reliability.

### 4.5. Measurement Error

The accuracy and reliability of the BIVA method are strongly dependent on proper calibration procedures, standardized measurement protocols, and the evaluation of methodological variability. Most studies reported routine calibration of analyzers, typically performed on a daily or weekly basis, using known impedance circuits. This practice is essential for ensuring measurement consistency and minimizing systematic errors that could compromise the validity of tolerance ellipses. To further assess the reliability of impedance measurements, several studies reported accuracy or error values, which underscored the technical reliability of the instruments used. Additionally, repeatability analyses, including assessments of intra- and inter-individual variability, were conducted in some studies. These analyses typically involved calculating coefficients of variation for within-day, between-day, and inter-observer measurements. A lower coefficient of variation reflects high measurement precision, thereby reducing the likelihood of random errors influencing the results. Another critical factor influencing measurement variability is the center effect, which was evaluated in only a few studies. The center effect accounts for variability introduced by both the operator (e.g., differences in electrode placement and measurement conditions) and the instrument (e.g., variations between different analyzers of the same model). Addressing these methodological considerations within the protocol is important for enhancing the robustness and applicability of BIVA in both clinical and sports settings.

### 4.6. Practical Implications

Our scoping review highlights several factors that should be carefully considered when applying or generating BIVA tolerance ellipses. For researchers aiming to develop new reference ellipses, it is essential to stratify the sample by age and BMI, ensure an adequate sample size to achieve robust and reliable data, and carefully standardize measurement procedures, including electrode placement, body position, and the type of analyzer used. In children and adolescent populations, stratification is particularly relevant due to the effects of biological maturation on vector length and orientation. Ethnicity was historically considered in the development of the Piccoli software (BIVA Software 2002) based on the hypothesis that it could influence bioelectrical values [98]. However, recent evidence indicates minimal effect when controlling for age, sex, and BMI, and ethnicity was retained in the software only to maintain methodological consistency [99]. Finally, it is crucial for researchers to report all methodological details that may influence bioelectrical variables, to increase study replicability and provide useful information for practitioners. For practitioners, including clinicians, nutritionists, and coaches, the focus should be on selecting the most appropriate reference ellipses for their population of interest. Differences in age, BMI, maturation stage, measurement protocols, and analyzer type can affect vector positioning, so comparisons should only be made with populations that closely match these characteristics. When such a match is not possible, developing cohort-specific ellipses is recommended to ensure accurate assessment of body composition and fluid distribution. Awareness of these factors enhances the applicability and reliability of BIVA for clinical, nutritional, and sports decision-making.

### 4.7. Strengths and Limits

The strengths of our scoping review include the use of a tested search strategy, which was sensitive enough to map and synthesize the topic and present the knowledge gaps. Although we used a sensitive search, it is possible that some studies were missed.

## 5. Conclusions

This scoping review included studies with heterogeneous methodologies, enabling the inclusion of research from diverse geographic regions and clinical or field-based settings. Such a broad approach has been instrumental in identifying key gaps and priorities for future investigation. Notably, based on the synthesis of available tolerance ellipses, there is a clear need to increase the number of ellipses available for pediatric and adolescent populations, with stratification by sex, age, BMI, and ethnicity. Similarly, certain pathological populations that could significantly benefit from BIVA in disease monitoring still lack appropriate reference ellipses. Within athletic populations, the availability of sport-specific ellipses remains limited, particularly for female athletes, who are underrepresented compared to their male counterparts. To enhance the quality and applicability of future reference ellipses, several methodological recommendations should be considered. Crucially, BIA analyzers are not cross-calibrated, meaning that measurements can vary significantly between devices, even when applied to the same individual under identical conditions [100]. Therefore, it is essential for future studies to explicitly report the brand and model of the analyzer used, along with details about electrode placement, configuration, and measurement protocol. Furthermore, the calibration procedure of the device should be transparently described to allow for replication and comparison across studies. The development of accurate and generalizable tolerance ellipses also requires the inclusion of larger, well-characterized sample sizes, representative of the target population. This review contributes to the field by cataloging existing tolerance ellipses based on population characteristics (sex, age, BMI), device specifications, and measurement protocols. It also compiles all the necessary bioelectrical data (sample size, R/H, Xc/H, and r) required for constructing reference ellipses. This comprehensive approach is intended to support researchers, clinicians, nutritionists, and sport scientists in selecting the most appropriate and methodologically sound tolerance ellipses for their specific populations of interest.

## Data Availability

All data generated or analyzed during this study are included in this published article (and its Supplementary Materials files).

## Supplementary Materials

The following supporting information can be downloaded at: https://www.mdpi.com/article/10.3390/jfmk10040415/s1, Table S1: PRISMA-ScR Checklist Item; Table S2: Search strategy in all databases; Table S3: List of studies excluded in the full-text screening phase; Table S4: Overview of included studies and their main characteristics; Table S5: General population: characteristics and values for tolerance ellipse construction; Table S6: Children–adolescent population: characteristics and values for tolerance ellipse construction; Table S7: Pathological population: characteristics and values for tolerance ellipse construction; Table S8: Athletic population: characteristics and values for tolerance ellipse construction.

## Abbreviations

The following abbreviations are used in this manuscript:

BIA	Bioelectrical impedance analysis
Z	Impedance
TBW	Total body water
R	Resistance
Xc	Reactance
PhA	Phase angle
FM	Fat mass
FFM	Fat-free mass
BIVA	Bioelectrical impedance vector analysis
ICW:ECW	Intracellular-to-extracellular water ratio
BCM	Body cellular mass
OSF	Open Science Framework
JBI	Joanna Briggs Institute
PRISMA-ScR	Preferred Reporting Items for Systematic Reviews and Meta-Analyses extension for Scoping Reviews
MeSH	Medical Subject Headings
BMI	Body mass index
*r*	Linear correlation coefficient
