# Supplementary material for: Reference Tolerance Ellipses in Bioelectrical Impedance Vector Analysis Across General, Pediatric, Pathological, and Athletic Populations: A Scoping Review
Source: J Funct Morphol Kinesiol. 2025 Oct 22;10(4):415. doi: 10.3390/jfmk10040415 (PMC12641658; doi:10.3390/jfmk10040415)
Supplement: Supplementary file 1 [file jfmk-10-00415-s001.zip › Supplementary Table S2.pdf]

Table S2. Search strategy in all databases.

Search date = 02/2025
**MEDLINE (PubMed); Recovered studies: 276**
("Impedance"[MeSH] OR "Electrical Impedance"[MeSH] OR "Bioelectrical Impedance"[MeSH] OR impedance[Text Word] OR electrical impedance[Text Word] OR bioelectrical impedance[Text Word] OR bioimpedance[Text Word] OR BIA[Text Word] OR Bio-electrical[Text Word]) **AND** (vector analysis[Text Word] OR bioelectrical impedance vector analysis[Text Word] OR impedance vector[Text Word] OR biavector[Text Word] OR BIA vector[Text Word] OR classical BIVA[Text Word] OR specific BIVA[Text Word] OR BIVA[Text Word] OR Bioelectrical impedance analysis[Text Word] OR specific bioelectrical impedance vector[Text Word] OR classical bioelectrical impedance vector[Text Word]) **AND** (ellipse*[Text Word] OR confidence ellipse*[Text Word] OR tolerance ellipse*[Text Word] OR R-Xc graph[Text Word] OR percentil*[Text Word] OR Bioimpedance Vector Reference*[Text Word])
**Cochrane Library; Recovered studies: 90**
#1 MeSH descriptor: [Electric Impedance] explode all trees
#2 bioimpendace (Word variations have been searched)
#3 impedance (Word variations have been searched)
#4 electrical impedance (Word variations have been searched)
#5 Bioelectrical Impedance (Word variations have been searched)
#6 bioimpedance (Word variations have been searched)
#7 Bio-electrical (Word variations have been searched)
#8 BIA (Word variations have been searched)
#9 {OR #1-#8} (Word variations have been searched)
#10 Vector Analysis
#11 bioelectrical impedance vector analysis
#12 impedance vector
#13 biavector
#14 BIA vector
#15 classical BIVA
#16 specific BIVA
#17 BIVA
#18 Bioelectrical impedance analysis (Word variations have been searched)
#19 specific bioelectrical impedance vector (Word variations have been searched)
#20 classical bioelectrical impedance vector (Word variations have been searched)
#21 {OR #10-#20}
#22 ellipse*
#23 confidence ellipse*
#24 tolerance ellipse*
#25 R-Xc graph
#26 percentil* (Word variations have been searched)
#27 Bioimpedance Vector Reference* (Word variations have been searched)
#28 {OR #22-#27}
#29 #9 AND #21 AND #28
**SPORTDiscus; Recovered studies: 31**
(impedance OR electrical impedance OR bioelectrical impedance OR bioimpedance OR BIA OR Bio-electrical) AND (vector analysis OR bioelectrical impedance vector analysis OR impedance vector OR biavector OR BIA vector OR classical BIVA OR specific BIVA OR BIVA OR Bioelectrical impedance analysis OR specific bioelectrical impedance vector OR classical bioelectrical impedance vector) AND (ellipse* OR confidence ellipse* OR tolerance ellipse* OR R-Xc graph OR percentil* OR Bioimpedance Vector Reference*)
**Scopus; Recovered Studies: 1980**
ALL (impedance OR "electrical impedance" OR "bioelectrical impedance" OR bioimpedance OR bia OR bio-electrical) AND ALL ("vector analysis" OR "bioelectrical impedance vector analysis" OR "impedance vector" OR biavector OR "BIA vector" OR "classical BIVA" OR "specific BIVA" OR biva OR "Bioelectrical impedance analysis" OR "specific bioelectrical impedance vector" OR "classical bioelectrical impedance vector") AND ALL (ellipse* OR "confidence ellipse*" OR "tolerance ellipse*" OR "R-Xc graph" OR percentil* OR "Bioimpedance Vector Reference*")
**Web of Science; Recovered studies: 362**
ALL=(impedance OR "electrical impedance" OR "bioelectrical impedance" OR bioimpedance OR BIA OR "bio-electrical") AND ALL=("vector analysis" OR "bioelectrical impedance vector analysis" OR "impedance vector" OR biavector OR "BIA vector" OR "classical BIVA" OR "specific BIVA" OR BIVA OR "bioelectrical impedance analysis" OR "specific bioelectrical impedance

vector" OR "classical bioelectrical impedance vector") AND ALL=(ellipse* OR "confidence ellipse*" OR "tolerance ellipse*" OR "R-Xc graph" OR percentil* OR "Bioimpedance Vector Reference*")
**Total studies: 2739**
