# Supplementary material for: Reference Tolerance Ellipses in Bioelectrical Impedance Vector Analysis Across General, Pediatric, Pathological, and Athletic Populations: A Scoping Review
Source: J Funct Morphol Kinesiol. 2025 Oct 22;10(4):415. doi: 10.3390/jfmk10040415 (PMC12641658; doi:10.3390/jfmk10040415)
Supplement: Supplementary file 1 [file jfmk-10-00415-s001.zip › Supplementary Table S3.pdf]

Table S3. List of studies excluded in the full-text screening phase.

AUTHOR	YEAR	TITLE	REASON
**Piccoli A. et al.** [1]	1996	Body Fluid Overload and Bioelectrical Impedance Analysis in Renal Patients	Essential information not reported
**Piccoli A. et al.** [21]	1996	A new method for monitoring hydration at high altitude by bioimpedance analysis	Essential information not reported
**Teodori T. et al** [101]	1997	Bioelectrical impedance analysis using graph RXc is not influenced by obesity in hemodialyzed patients: Preliminary results; [L'obesita non introduce distorsioni nell'analisi di bioimpedenza con grafo RXc dell'uremico cronico in emodialisi: Risultati preliminari]	Article in a non-eligible language
**Piccoli A. et al.** [102]	1997	Asymmetry of the total body water prediction bias using the impedance index	The study does not fit the research criteria
**Di Iorio B. et al.** [103]	1998	Total Body Water and Body Cell Mass in Normal Weight Healthy Adults	The study does not fit the research criteria
**Guglielmi F.W. et al.** [104]	1998	Utility of RXc graph in evaluating altered fluid balance in patients with chronic liver disease; [Utilita del grafico RXc nella valutazione dello scompenso idrico dell'epatopatia cronica]	Article in a non-eligible language
**Guida B. et al.** [105]	2000	Comparison of vector and conventional bioelectrical impedance analysis in the optimal dry weight prescription in hemodialysis	Essential information not reported
**Piccoli A. et al.** [106]	2000	Relationship between central venous pressure and bioimpedance vector analysis in critically ill patients	The study does not fit the research criteria
**Savino F. et al.** [107]	2004	The biagram vector: A graphical relation between reactance and phase angle measured by bioelectrical analysis in infants	The study does not fit the research criteria
**Barbosa-Silva M.C.G. et al.** [108]	2005	Bioelectrical impedance analysis in clinical practice: A new perspective on its use beyond body composition equations	Wrong study desing
**Jha V. et al.** [109]	2006	Body composition analysis with bioelectric impedance in adult Indians with ESRD: Comparison with healthy population	Essential information not reported
**Espinosa-Cuevas M. de L.A. et al.** [110]	2007	Bioimpedance vector analysis for body composition in Mexican population; [Vectores de impedancia bioeléctrica para la composición corporal en población mexicana]	Article in a non-eligible language
**Guida B. et al.** [111]	2007	Bioelectrical impedance analysis and age-related differences of body composition in the elderly	Essential information not reported
**Buffa R. et al.** [112]	2009	Bioelectrical impedance vector analysis in the assessment of nutritional status in the elderly	Essential information not reported
**Buffa R. et al.** [113]	2009	Assessment of nutritional status in free-living elderly individuals by bioelectrical impedance vector analysis	The study does not fit the research criteria
**Stobäus N. et al.** [114]	2010	Phase angle and bioelectrical impedance vector analysis clinical practicability of impedance parameters; [Phasenwinkel und bioelektrische impedanzvektoranalyse klinische anwendbarkeit der impedanzparameter]	Article in a non-eligible language
**Bozzetto S. et al.** [115]	2010	Bioelectrical impedance vector analysis to evaluate relative hydration status	The study does not fit the research criteria

**Alvero-Cruz J.R. et al.** [116]	2011	Bioelectrical impedance analysis as a method of body composition estimation: A practical approach; [La bioimpedancia eléctrica como método de estimación de la composición corporal: Normas prácticas de utilización]	Article in a non-eligible language
**Santomauro F. et al.** [117]	2011	Bioelectrical impedance vector analysis and mini nutritional assessment in elderly nursing home residents	Essential information not reported
**Lukaski H.C. et al.** [118]	2012	Bioelectrical impedance vector analysis for assessment of hydration in physiological states and clinical conditions	Wrong study desing
**Centellas Tristán M.T. et al.** [119]	2013	Assessing dry weight and body water using bioimpedance vector analysis compared to the traditional method; [evaluación del peso seco y el agua corporal según bioimpedancia vectorial frente al método tradicional]	Article in a non-eligible language
**Koury J.C. et al.** [120]	2014	Phase angle and bioelectrical impedance vectors in adolescent and adult male athletes	Essential information not reported
**Camina Martín M.A. et al.** [121]	2014	Specific bioelectrical impedance vector analysis (BIVA) is more accurate than classic BIVA to detect changes in body composition and in nutritional status in institutionalised elderly with dementia	Essential information not reported
**Piccoli A. et al.** [122]	2014	Combined evaluation of nutrition and hydration in dialysis patients with bioelectrical impedance vector analysis (BIVA)	The study does not fit the research criteria
**Atilano-Carsi X. et al.** [123]	2015	Normal values of bioimpedance vector in Spanish population; [Vectores de impedancia bioeléctrica de referencia para la población española]	Article in a non-eligible language
**Pigłowska M. et al.** [124]	2015	Bioelectrical impedance vector analysis as an auxiliary method in diagnosing of sarcopenia among hospitalized older patients - A preliminary report	Essential information not reported
**Camina Martín M.A. et al.** [125]	2015	Bioimpedance vector analysis and conventional bioimpedance to assess body composition in older adults with dementia	Essential information not reported
**Alves F.D. et al.** [126]	2015	Dynamic changes in bioelectrical impedance vector analysis and phase angle in acute decompensated heart failure	The study does not fit the research criteria
**Klimenko A. et al.** [127]	2015	Prognostic value of bioimpedance vector analysis versus clinical characteristics in patients with acute decompensation of heart failure	The study does not fit the research criteria
**Massari F. et al.** [128]	2016	Accuracy of bioimpedance vector analysis and brain natriuretic peptide in detection of peripheral edema in acute and chronic heart failure	Essential information not reported
**Khalil S.F. et al.** [129]	2016	Bioimpedance vector analysis in diagnosing severe and non-severe dengue patients	Essential information not reported
**Redondo-del-Río M.P. et al.** [130]	2016	Vector bioimpedance detects situations of malnutrition not identified by the indicators commonly used in geriatric nutritional assessment: A pilot study	Essential information not reported
**Heavens K.R. et al.** [131]	2016	Noninvasive assessment of extracellular and intracellular dehydration in healthy humans using the resistance-reactance-score graph method	The study does not fit the research criteria
**Veitia W.C. et al.** [132]	2017	Body composition analysis using bioelectrical parameters in Cuban sporting population; [Análisis de la composición corporal empleando parámetros bioeléctricos en la población deportiva cubana]	Article in a non-eligible language
**Cardoso I.C.R. et al.** [133]	2017	Applicability of the direct parameters of bioelectrical impedance in assessing nutritional status and surgical complications of women with gynecological cancer	Essential information not reported

**Carrasco-Marginet M. et al.** [91]	2017	Bioelectrical impedance vector analysis (BIVA) for measuring the hydration status in young elite synchronized swimmers	Essential information not reported
**Meleleo D. et al.** [134]	2017	Evaluation of body composition with bioimpedence. A comparison between athletic and non-athletic children	Essential information not reported
**Buffa R. et al.** [135]	2017	Specific BIVA recognizes variation of body mass and body composition: Two related but different facets of nutritional status	Essential information not reported
**Dehesa-López E. et al.** [136]	2017	Discordance between bioelectrical impedance vector analysis and the new ESPEN definition of malnutrition for the diagnosis of hospital malnutrition	The study does not fit the research criteria
**Hise A.C.D.R. et al.** [137]	2018	Assessment of hydration status using bioelectrical impedance vector analysis in critical patients with acute kidney injury	Essential information not reported
**Santillán-Díaz C. et al.** [138]	2018	Prevalence of rheumatoid cachexia assessed by bioelectrical impedance vector analysis and its relation with physical function	Essential information not reported
**Pineda-Juárez J.A. et al.** [139]	2018	Body composition evaluated by body mass index and bioelectrical impedance vector analysis in women with rheumatoid arthritis	The study does not fit the research criteria
**Castizo-Olier J. et al.** [140]	2018	Bioelectrical impedance vector analysis (BIVA) in sport and exercise: Systematic review and future perspectives	Wrong study desing
**Limon-Miro A.T. et al.** [141]	2019	Bioelectric impedance vector analysis (Biva) in breast cancer patients: A tool for research and clinical practice	Essential information not reported
**Vermeulen K.M. et al.** [142]	2019	Bioelectrical impedance vector analysis and phase angle in boys with duchenne muscular dystrophy	Essential information not reported
**De-Mateo-Silleras B. et al.** [143]	2019	Bioelectrical impedance vector analysis in obese and overweight children	The study does not fit the research criteria
**Campa F. et al.** [144]	2020	Somatotype and bioimpedance vector analysis: A new target zone for male athletes	Essential information not reported
**Oliveira Filho J.M. et al.** [145]	2020	Bioelectrical vector analysis in obese adolescents	Essential information not reported
**Reis J.F. et al.** [146]	2020	Bioimpedance vector patterns changes in response to swimming training: An ecological approach	Essential information not reported
**Campa F. et al.** [147]	2020	Body water content and morphological characteristics modify bioimpedance vector patterns in volleyball, soccer, and rugby players	Essential information not reported
**Fernandes S.A. et al.** [148]	2020	Bioelectrical impedance vector analysis evaluates cellularity and hydration in cirrhotic patients	The study does not fit the research criteria
**Shchelykalina S.P. et al.** [149]	2020	Data views technology of bioimpedance vector analysis of human body composition	The study does not fit the research criteria
**Fernandes S.A. et al.** [150]	2020	Retrospective Study: Bioelectrical impedance vector analysis evaluates cellularity and hydration in cirrhotic patients	The study does not fit the research criteria
**Almeida Y.L. et al.** [151]	2021	Is bioelectrical impedance vector analysis a good indicator of nutritional status in children and adolescents?	Essential information not reported
**Lu H.-K. et al.** [152]	2021	Assessment of total and regional bone mineral density using bioelectrical impedance vector analysis in elderly population	Essential information not reported
**Martins P.C. et al.** [153]	2021	Bioelectrical impedance vector analysis (BIVA) in university athletes	Essential information not reported

**Machado V.M.Q. et al.** [154]	2021	Bioelectrical impedance vector applied to body composition evaluation of women survivors of breast cancer: A longitudinal study	Essential information not reported
**Regli I.B. et al.** [155]	2021	Bioelectrical impedance vector analysis: A valuable tool to monitor daily body hydration dynamics at altitude	The study does not fit the research criteria
**Costa D. et al.** [156]	2021	Fluid Status After Cardiac Surgery Assessed by Bioelectrical Impedance Vector Analysis and the Effects of Extracorporeal Circulation	The study does not fit the research criteria
**Girma T. et al.** [157]	2021	Utility of bio-electrical impedance vector analysis for monitoring treatment of severe acute malnutrition in children	The study does not fit the research criteria
**Campa F. et al.** [158]	2021	Leucine metabolites do not induce changes in phase angle, bioimpedance vector analysis patterns, and strength in resistance trained men	The study does not fit the research criteria
**Roccamatisi L. et al.** [159]	2021	Preoperative standardized phase angle at bioimpedance vector analysis predicts the outbreak of antimicrobial-resistant infections after major abdominal oncologic surgery: A prospective trial	The study does not fit the research criteria
**De la Cruz Marcos S. et al.** [160]	2021	Applications of bioelectrical impedance vector analysis (Biva) in the study of body composition in athletes	Wrong study desing
**Campa F. et al.** [161]	2021	Assessment of body composition in athletes: A narrative review of available methods with special reference to quantitative and qualitative bioimpedance analysis	Wrong study desing
**Miranda-Alatriste P.V. et al.** [162]	2022	Hydration status according to impedance vectors and its association with clinical and biochemical outcomes and mortality in patients with chronic kidney disease; [Estado de hidratación por vectores de impedancia y su asociación con desenlaces clínicos, bioquímicos y mortalidad en pacientes con enfermedad renal crónica]	Article in a non-eligible language
**Grusdat N.P. et al.** [163]	2022	A Prospective Observational Pilot Study of Young Women Undergoing Initial Breast Cancer Treatment and Their Biopsychosocial Profile	Essential information not reported
**Rashid Farokhi F. et al.** [164]	2022	Applying bio-impedance vector analysis (BIVA) to adjust ultrafiltration rate in critically ill patients on continuous renal replacement therapy: a randomized controlled trial	Essential information not reported
**Bauermann A. et al.** [165]	2022	Bioelectrical impedance vector analysis and body composition in cervical spinal cord injury: A pilot study	The study does not fit the research criteria
**Matias C.N. et al.** [166]	2022	Bioelectrical Impedance Vector Analysis Discriminates Aerobic Power in Futsal Players: The Role of Body Composition	The study does not fit the research criteria
**Santos B.C. et al.** [167]	2022	Bioelectrical impedance vector analysis in patients on the waiting list for liver transplant: Associated factors and prognostic effects	The study does not fit the research criteria
**Castillo-Martínez L. et al.** [168]	2022	Evaluation of Fluid Overload by Bioelectrical Impedance Vectorial Analysis	The study does not fit the research criteria
**Silleras B.D.M. et al.** [169]	2023	Bioelectrical Impedance Vector Analysis (BIVA) and Somatotype in Female Rugby Players	Essential information not reported
**Vermeulen-Serpa K.M. et al.** [170]	2023	Gender-specific bioelectrical impedance reference values in healthy children	Essential information not reported
**Juan Antonio P.-J. et al.** [171]	2023	Evaluation of Hydration Status by Bioelectrical Impedance Vector Analysis in Patients with Ischemic Heart Disease Undergoing Exercise Stress Test	Essential information not reported
**Stagi S. et al.** [172]	2023	Body Composition and Strength Symmetry of Kettlebell Sport Athletes	The study does not fit the research criteria

**Caparello G. et al.** [173]	2023	Evaluation of Body Composition Changes by Bioelectrical Impedance Vector Analysis in Volleyball Athletes Following Mediterranean Diet Recommendations during Italian Championship: A Pilot Study	The study does not fit the research criteria
**Silva A.M. et al.** [174]	2023	The bioelectrical impedance analysis (BIA) international database: aims, scope, and call for data	The study does not fit the research criteria
**Bernal-Ceballos F. et al.** [175]	2023	Clinical Application of Phase Angle and BIVA Z-Score Analyses in Patients Admitted to an Emergency Department with Acute Heart Failure	The study does not fit the research criteria
**Díaz Rincón M. et al.** [176]	2024	Bioelectrical impedance vectors in the Colombian university population; [Vectores de impedancia bioeléctrica en la población universitaria colombiana]	Article in a non-eligible language
**Cebrián-Ponce Á. et al.** [177]	2024	Bioelectrical impedance vector analysis and track and field jump performance across different specialties: Sex differences and electrode configuration	Essential information not reported
**Marini E. et al.** [178]	2024	Specific bioelectrical vectors pattern in individuals with sarcopenic obesity	Essential information not reported
**Marini E. et al.** [179]	2024	Associations of bioelectrical impedance and anthropometric variables among populations and within the full spectrum of malnutrition	The study does not fit the research criteria
**Bertozzi F. et al.** [180]	2024	Bioimpedance Vector Analysis-Derived Body Composition Influences Strength and Power in Alpine Skiers	The study does not fit the research criteria
**Cattem M.V.D.O. et al.** [181]	2024	One-Year Changes in Bioelectrical Impedance Data in Adolescent Athletes	The study does not fit the research criteria
**Cunha T.A. et al.** [182]	2024	Phase Angle and Bioelectrical Impedance Vector Analysis (BIVA) in Amyotrophic Lateral Sclerosis (ALS) Patients	The study does not fit the research criteria
**Pedro da Costa Pereira J. et al.** [183]	2024	Prognostic value of overhydration and bioelectrical impedance vector on short- and long-term outcomes in hospitalized patients with cancer	The study does not fit the research criteria
**Cebrián-Ponce A. et al.** [184]	2024	Assessment of body composition and bioimpedance in runners with Down syndrome: insights derived from a four-case study	Wrong study desing
**Serafini S. et al.** [92]	2025	Hydration in young water polo players: A bioelectrical impedance vector analysis (BIVA) approach	Essential information not reported
