# Supplementary material for: Reference Tolerance Ellipses in Bioelectrical Impedance Vector Analysis Across General, Pediatric, Pathological, and Athletic Populations: A Scoping Review
Source: J Funct Morphol Kinesiol. 2025 Oct 22;10(4):415. doi: 10.3390/jfmk10040415 (PMC12641658; doi:10.3390/jfmk10040415)
Supplement: Supplementary file 1 [file jfmk-10-00415-s001.zip › Supplementary Table S4.pdf]

Table S4. Overview of included studies and their main characteristics.

AUTHOR	YEAR	TITLE	COUNTRY	POPULATION	ELLIPSES	ANALYZER	BODY SIDE	ERROR
**Stolarczyk LM**	**1994**	Predictive accuracy of bioelectrical impedance in estimating body composition of Native American women [34]	United States	General	1 (Classical BIVA)	Model 1990B (Valhalla Scientific, San Diego, CA, USA) at 50 KHz	Supine position. Two electrodes were placed on the right wrist and two on the right ankle, with at least 5 cm spacing.	n.s.
**Piccoli A.**	**1995**	Bivariate normal values of the bioelectrical impedance vector in adult and elderly populations [35]	Italy	General	2 (Classical BIVA)	BIA-109 (Akern Srl, Florence, Italy) at 50 kHz	Supine position. Two electrodes were placed on the right wrist and two on the right ankle, with at least 5 cm spacing.	The center effect (ie, the sum of operator and instrument variabilities), expressed as a CV, was 3% for R and 8% for Xc.
**Roubenoff R.**	**1997**	Application of bioelectrical impedance analysis to elderly populations [36]	United States	General	4 (Classical BIVA)	BIA-101 (Akern Srl, Florence, Italy) at 50 KHz; BCA analyzer (BCA Inc., Schaumberg, IL) at 50 KHz	Supine position. Two electrodes were placed on the right wrist and two on the right ankle, with at least 5 cm spacing.	n.s.
**Piccoli A.**	**1998**	Identification of operational clues to dry weight prescription in hemodialysis using bioimpedance vector analysis [38]	Italy	Pathological	4 (Classical BIVA)	BIA-101 (Akern Srl, Florence, Italy) at 50 KHz	Supine position. Two electrodes were placed on the right wrist and two on the right ankle, with at least 5 cm spacing.	n.s.
**Piccoli A.**	**1998**	Discriminating between body fat and fluid changes in the obese adult using bioimpedance vector analysis [39]	Italy	Pathological	4 (Classical BIVA)	BIA-109 (Akern Srl, Florence, Italy) at 50 kHz	Supine position. Two electrodes were placed on the right wrist and two on the right ankle, with at least 5 cm spacing.	Calibration: known impedance circuit (R = 470Ω, Xc = 90Ω, 1% error). Mean CV: 1% (within-day), 3% (weekly), 2% (interoperator).
**Guglielmi F.W.**	**1999**	The RXc graph in evaluating and monitoring fluid balance in patients with liver cirrhosis [40]	Italy	Pathological	8 (Classical BIVA)	BIA-101 (Akern Srl, Florence, Italy) at 50 KHz	Supine position. Two electrodes were placed on the right wrist and two on the right ankle, with at least 5 cm spacing.	n.s.

**De Palo T.**	**2000**	Normal values of the bioelectrical impedance vector in childhood and puberty [26]	Italy	Children-adolescents	10 (Classical BIVA)	BIA-101 (Akern Srl, Florence, Italy) at 50 KHz	Supine position. Two electrodes were placed on the right wrist and two on the right ankle, with at least 5 cm spacing.	Calibrated with known impedance (R = 470Ω, Xc = 90Ω, 1% error). Within-operator CV: 0.2% (R), 0.5% (Xc). Center effect CV: 3% (R), 8% (Xc).
**Toso S.**	**2000**	Altered tissue electrical properties in lung cancer patients detected by bioelectrical impedance vector analysis [41]	Italy	Pathological	2 (Classical BIVA)	BIA-101 (Akern Srl, Florence, Italy) at 50 KHz	Supine position. Two electrodes were placed on the right wrist and two on the right ankle, with at least 5 cm spacing.	Calibrated before each session with known impedance circuit (R = 470Ω, Xc = 90Ω, 1% error).
**Piccoli A.**	**2002**	Reference values of the bioelectrical impedance vector in neonates in the first week after birth [42]	Italy	Children-adolescents	4 (Classical BIVA)	BIA-101 (Akern Srl, Florence, Italy) at 50 KHz	Supine position. Two electrodes were placed on the right wrist and two on the right ankle, with at least 5 cm spacing.	Calibration was checked before each session. Test-retest and interobserver studies were conducted on 10 neonates. Within-observer CVs: 1.6% (R), 7.3% (Xc). Interobserver CVs: 2.6% (R), 7.8% (Xc)
**Piccoli A.**	**2002**	Impedance vector distribution by sex, race, body mass index, and age in the United States: Standard reference intervals as bivariate Z scores [37]	Italy, United States	General	108 (Classical BIVA)	Model 1990B (Valhalla Scientific, San Diego, CA, USA) at 50 KHz	Supine position. Two electrodes were placed on the right wrist and two on the right ankle, with at least 5 cm spacing.	n.s.
**Bogonez P.**	**2003**	Bioelectrical Impedance Vector Analysis in COPD Patients [29]	Spain	General; Pathological	1 (Classical BIVA);1 (Classical BIVA	Quantum Analyser (RJL Systems, Clinton Township, MI), at 50 kHz	Supine position. One electrode pair was placed at the right hand joint (5 cm separation) and the other at the right ankle joint (6 cm separation).	n.s.
**Savino F.**	**2003**	Bioelectrical impedance vector distribution in the first year of life [43]	Italy	Children-adolescents	12 (Classical BIVA)	STA/BIA (Akern Srl, Florence, Italy), at 50 kHz	Supine position. Two electrodes were placed on the right wrist and two on the right ankle, with at least 5 cm spacing.	The external calibration of the instrument was checked with a calibration circuit of

								known impedance value (R 380 and Xc 47, 1% error).
**Nescolarde L.**	**2004**	Bioelectrical impedance vector analysis in haemodialysis patients: Relation between oedema and mortality [50]	Cuba	General; Pathological	2 (Classical BIVA); 4 (Classical BIVA)	Bioscan xBL-960141 (Maltron International Ltd, Essex, UK)	Supine position. Two electrodes were placed on the right wrist and two on the right ankle, with at least 5 cm spacing.	Measurement errors are lower than 1 Ohm and 1◦ at 50 kHz using electrical models
**Piccoli A.**	**2004**	Bioelectric impedance vector distribution in peritoneal dialysis patients with different hydration status [53]	Italy	Pathological	4 (Classical BIVA)	BIA-101 (Akern Srl, Florence, Italy) at 50 KHz	Supine position. Two electrodes were placed on the right wrist and two on the right ankle, with at least 5 cm spacing.	The external calibration of the instrument was checked with a calibration circuit of known impedance value (R = 470 ohm, and Xc = 90 ohm, error 1%)
**Bosy-Westphal A.**	**2005**	Patterns of bioelectrical impedance vector distribution by body mass index and age: Implications for body-composition analysis [44]	Germany	General; Children-adolescents	70 (Classical BIVA); 28 (Classical BIVA)	BIA 2000-S (Data Input, Frankfurt, Germany) at 50 KHz	Supine position. Two electrodes were placed on the right wrist and two on the right ankle, with at least 5 cm spacing.	Mean CVs: Within-day <2% (R), <3.5% (Xc); Between-day <1.5% (R), <5% (Xc).
**Lukaski H.C.**	**2007**	Assessment of change in hydration in women during pregnancy and postpartum with bioelectrical impedance vectors [51]	United States	General	5 (Classical BIVA)	BIA-101 (Akern Srl, Florence, Italy) at 50 KHz	Supine position. Two electrodes were placed on the right wrist and two on the right ankle, with at least 5 cm spacing.	Calibrated before each session with known impedance circuit (R = 450 and Xc = 65), was 1%. The mean coefficient of variation was 1% for within-day and 2% for between-day intraindividual measurements determined in another group.
**Guida B.**	**2008**	Body mass index and bioelectrical vector distribution in 8-year-old children [45]	Italy	Children-adolescents	4 (Classical BIVA)	BIA-101 (Akern Srl, Florence, Italy) at 50 KHz	Supine position. Two electrodes were placed on the right wrist and two on	n.s.

							the right ankle, with at least 5 cm spacing.	
**Margutti A.V.B.**	**2010**	Reference distribution of the bioelectrical impedance vector in healthy term newborns [46]	Brazil	Children-adolescents	3 (Classical BIVA)	Quantum II Analyzer (RJL Systems, Clinton Township, MI)	Supine position. Adhesive electrodes were placed on the right wrist and two on the right ankle.	n.s.
**L'Abée C.**	**2010**	The bioelectrical impedance vector migration in healthy infants [47]	Netherlands	Children-adolescents	3 (Classical BIVA)	BIA-101 (Akern Srl, Florence, Italy) at 50 KHz	Supine position. Two electrodes were placed on the right wrist and two on the right ankle, with at least 5 cm spacing.	n.s.
**Tanabe R.F.**	**2012**	Distribution of bioelectrical impedance vector values in multi-ethnic infants and pre-school children [48]	Brazil	Children-adolescents	5 (Classical BIVA)	Xitron Hydra ECF/ICF Bio-Impedance Analyzer System Model 4200 (Xitron Technologies, San Diego, CA)	Supine position. Two electrodes were placed on the right wrist and two on the right ankle, with at least 5 cm spacing.	n.s.
**Buffa R.**	**2013**	Accuracy of Specific BIVA for the Assessment of Body Composition in the United States Population [25]	United States	General	1 (Classical BIVA); 3 (Specific BIVA)	Xitron Hydra ECF/ICF Bio-Impedance Analyzer System Model 4200 (Xitron Technologies, San Diego, CA)	Supine position. Two electrodes were placed on the right wrist and two on the right ankle, with at least 5 cm spacing.	n.s.
**Nescolarde L.**	**2013**	Reference values of the bioimpedance vector components in a Caribbean population [49]	Cuba	General; Children-adolescents	4 Classical BIVA); 12 (Classical BIVA)	Bioscan xBL-960141 (Maltron International Ltd, Essex, UK)	Supine position. Two electrodes were placed on the right wrist and two on the right ankle, with at least 5 cm spacing.	n.s.
**Siváková D.**	**2013**	Bioelectrical Impedance Vector Analysis (BIVA) in Slovak population: Application in a clinical sample [52]	Slovakia	General; Pathological	14 (Classical BIVA); 9 (Classical BIVA)	BIA-101 (Akern Srl, Florence, Italy) at 50 KHz	Supine position. Two electrodes were placed on the right wrist and two on the right ankle, with at least 5 cm spacing.	n.s.
**Micheli M.L.**	**2014**	Bioimpedance and impedance vector patterns as predictors of league level in male soccer players [54]	Italy	Athletes	6 (Classical BIVA)	BIA-101 (Akern Srl, Florence, Italy) at 50 KHz	Supine position. Two electrodes were placed on the right wrist and two on the right ankle, with at least 5 cm spacing.	Calibrated daily with a known impedance circuit (R = 380Ω, Xc = 47Ω, 1% error).

**Saragat B.**	**2014**	Specific bioelectrical impedance vector reference values for assessing body composition in the Italian elderly [27]	Italy	General	2 (Classical BIVA); 2 (Specific BIVA)	BIA-101 (Akern Srl, Florence, Italy) at 50 KHz	Supine position. Two electrodes were placed on the right wrist and two on the right ankle, with at least 5 cm spacing.	n.s.
**Mathias-Genovez M.G.**	**2015**	Bioelectrical Impedance of Vectorial Analysis and Phase Angle in Adolescents [71]	Brazil	Children-adolescents	18 (Classical BIVA)	BIA 103-A (RJL Systems, Clinton Township, MI), at 800 mA	Supine position. Two electrodes were placed on the dorsal surface of the hands and two on the surface of the feet	n.s.
**Ibáñez M.E.**	**2015**	New specific bioelectrical impedance vector reference values for assessing body composition in the Italian-Spanish young adult population [65]	Italy, Spain	General	2 (Specific BIVA)	BIA-101 (Akern Srl, Florence, Italy) at 50 KHz	Supine position. Two electrodes were placed on the right wrist and two on the right ankle, with at least 5 cm spacing.	Accuracy: 1 Ohm
**Redondo-del-Río M.P.**	**2017**	Bioelectrical impedance vector reference values for assessing body composition in a Spanish child and adolescent population [73]	Spain	Children-adolescents	24 (Classical BIVA)	RJL 101-A (RJL Systems, Clinton Township, MI), at 50 kHz	Supine position .Electrodes were placed on the dorsum of both the hand and foot of the dominant side of the body	measurement errors of <1% for R and <2% for capacitance, as assessed using a precision resistor and capacitor
**Toffano R.B.D.**	**2017**	Bioelectrical Impedance Vector Analysis in Healthy Term Infants in the First Three Months of Life in Brazil [72]	Brazil	Children-adolescents	3 (Classical BIVA)	Quantum II (RJL Systems, Clinton Township, MI)	Supine position. Two outer electrodes were placed on the dorsal surfaces of the right hand and foot, and two inner electrodes on the right forearm and pretibial region, with 5.5 cm of free skin around the outer electrodes.	n.s.
**Castizo-Olier J.**	**2018**	Bioelectrical impedance vector analysis (BIVA) and body mass changes in an ultra-endurance triathlon event [55]	Spain	Athletes	1 (Classical BIVA)	Z-Métrix® (BioparHom®, Bourget du Lac, France) at 77 µA alternating sinusoidal current at frequencies from 1 to 325 kHz	Position not specified. Two electrodes were placed on the right wrist and two on the right ankle, with at least 5 cm spacing.	The device provides impedance values with an accuracy characterized by an average error of 0.95% ± 1.58% and an average repeatability errors of 0.55% ±

								0.38% for all the frequency range
**Campa F.**	**2018**	Bioimpedance Vector Analysis of Elite, Subelite, and Low-Level Male Volleyball Players [56]	Italy	Athletes	4 (Classical BIVA)	BIA 101 Anniversary (Akern Srl, Florence, Italy), at 50 kHz	Supine position. Two electrodes were placed on the right wrist and two on the right ankle, with at least 5 cm spacing.	n.s.
**Giorgi A.**	**2018**	Bioimpedance patterns and bioelectrical impedance vector analysis (BIVA) of road cyclists [57]	Italy	Athletes	6 (Classical BIVA)	BIA-101 (Akern Srl, Florence, Italy) at 50 KHz	Supine position. Two electrodes were placed on the right wrist and two on the right ankle, with at least 5 cm spacing.	Instrument accuracy: 1% (R), 1% (Xc), verified daily with a calibration circuit (R = 380Ω, Xc = 47Ω)
**Koury J.C.**	**2018**	Bioimpedance parameters in adolescent athletes in relation to bone maturity and biochemical zinc indices [58]	Brazil	Athletes	3 (Classical BIVA)	Model 101 Quantum (RJL Systems, Clinton Township, MI) at 50 KHz	Supine position. Two electrodes were placed on the right wrist and two on the right ankle, with at least 5 cm spacing.	Calibrated before each session with known impedance circuit (resistance = 500.0 ohms; reactance = 0.1 ohms, 0.9% error)
**Jensen B.**	**2019**	Ethnic differences in fat and muscle mass and their implication for interpretation of bioelectrical impedance vector analysis [66]	Germany, Japan, Mexico	General	48 (Classical BIVA)	Seca mBCA 514 (Seca GmbH & Co. KG, Hamburg, Germany)	Orthostatic position. Each side of the ascending handrail had 6 electrodes, with 2 selected based on height, requiring an upright stance with outstretched arms. Two electrode pairs were placed on the feet, enabling segmental impedance measurement with an 8-electrode technique.	The accuracy of measurements ofthe right and left body side at frequencies of5 and 50 kHz is 5 Ohm for the impedance and 0.5° for the phase angle.
**Oh J.-H.**	**2019**	Normal reference plots for the bioelectrical impedance vector in healthy Korean adults [67]	Korea	General	2 (Classical BIVA)	InBody S10® (InBody Co.,Ltd., Seoul, Korea)	Supine position. Tetra-polar eight-point tactile electrode system	n.s.

**Campa F.**	**2019**	Classic bioelectrical impedance vector reference values for assessing body composition in male and female athletes [28]	Italy	Athletes	8 (Classical BIVA)	BIA 101 Anniversary (Akern Srl, Florence, Italy), at 50 kHz	Supine position. Two electrodes were placed on the right wrist and two on the right ankle, with at least 5 cm spacing.	n.s.
**Redondo-del-Río M.P.**	**2019**	Bioelectrical impedance vector values in a Spanish healthy newborn population for nutritional assessment [74]	Spain	Children-adolescents	3 (Classical BIVA)	BIA-101 (Akern Srl, Florence, Italy) at 50 KHz	Supine position. Two electrodes were placed on the right wrist and two on the right ankle, with at least 5 cm spacing.	n.s.
**Bongiovanni T.**	**2020**	Bioimpedance vector references need to be period-specific for assessing body composition and cellular health in elite soccer players: A brief report [61]	Italy	Athletes	1 (Classical BIVA)	BIA 101 Anniversary (Akern Srl, Florence, Italy), at 50 kHz	Supine position. Two electrodes were placed on the right wrist and two on the right ankle, with at least 5 cm spacing.	n.s.
**Marini E.**	**2020**	Phase angle and bioelectrical impedance vector analysis in the evaluation of body composition in athletes [23]	Italy	Athletes	2 (Classical BIVA); 2 (Specific BIVA)	BIA 101 Anniversary (Akern Srl, Florence, Italy), at 50 kHz	Supine position. Two electrodes were placed on the right wrist and two on the right ankle, with at least 5 cm spacing.	Test-retest CVs: 0.3% (R), 0.9% (Xc). Reference: Italo-Spanish bioelectrical specific values. Classic values (unpublished): R/H (men: 284.9 ± 33.6, women: 391.2 ± 41.1); Xc/H (men: 38.0 ± 5.0, women: 44.0 ± 5.8).
**Nescolarde L.**	**2020**	Relationship Between Bioimpedance Vector Displacement and Renal Function After a Marathon in Non-elite Runners [60]	Spain	Athletes	1 (Classical BIVA)	BIA 101 Anniversary (Akern Srl, Florence, Italy), at 50 kHz	Supine position. Two electrodes were placed on the right wrist and two on the right ankle, with at least 5 cm spacing.	The measurement or technical errors of the system, determined with a parallel circuit of precision resistor and capacitor, were <1 Ohm for R and <2% for capacitance.
**Toselli S.**	**2020**	Maturity related differences in body composition assessed by classic and specific bioimpedance vector analysis among male elite youth soccer players [59]	Italy	Athletes	1 (Classical BIVA); 1 (Specific BIVA)	BIA 101 Anniversary (Akern Srl, Florence, Italy), at 50 kHz	Supine position. Two electrodes were placed on the right wrist and two on the right ankle, with at least 5 cm spacing.	n.s.

**Gomes T.L.M.**	**2021**	Association of electrical bioimpedance vectors with the nutritional classification of vulnerable multiethnic children [75]	Brazil	Children-adolescents	5 (Classical BIVA)	Xitron Hydra ECF/ICF Bio-Impedance Analyzer System Model 4200 (Xitron Technologies, San Diego, CA)	Supine position. Sensor electrodes were placed on the dorsal surface of the wrist (between the ulna and radius) and the anterior surface of the ankle (between the protruding bone portions), while injector electrodes were positioned on the dorsal surface of the third proximal phalanx of the hand and foot.	n.s.
**Di Credico A.**	**2021**	Bioelectrical impedance vector analysis of young elite team handball players [62]	Italy	Athletes	2 (Classical BIVA)	BIA 101 Anniversary (Akern Srl, Florence, Italy), at 50 kHz	Supine position. Two electrodes were placed on the right wrist and two on the right ankle, with at least 5 cm spacing.	n.s.
**Rossini-Venturini A.C.**	**2022**	Association between classic and specific bioimpedance vector analysis and sarcopenia in older adults: a cross-sectional study [68]	Brazil	General; Pathological	2 (Classical BIVA) and 2 (Specif BIVA); 2 (Classical BIVA) and 2 (Specif BIVA)	BIA Imp DF50 Body Composition Analyzer (ImpediMed, Brisbane, Queensland, Australia)	Supine position. Two electrodes were placed on the right wrist and two on the right ankle, with at least 5 cm spacing.	n.s.
**Campa F.**	**2023**	New bioelectrical impedance vector references and phase angle centile curves in 4,367 adults: The need for an urgent update after 30 years [69]	Italy	General	2 (Classical BIVA)	BIA 101 Anniversary (Akern Srl, Florence, Italy), at 50 kHz; BIA 101 BIVA PRO (Akern Srl, Florence, Italy), at 50 kHz	Supine position. Two electrodes were placed on the right wrist and two on the right ankle, with at least 5 cm spacing.	The accuracy of theanalyzers was verified using a reference circuit with acceptance for R measurements of 383 ohm and Xc values of 46 U; the test-retest coefficient of variation (CV% = standard deviation/mean*100%) on duplicate measurements of R and Xc was 0.3% and 0.9%, respectively.

**Izzicupo P.**	**2023**	Morphological Characteristics of Elite International Soccer Referees: Somatotype and Bioelectrical Impedance Vector Analysis [63]	Italy	Athletes	1 (Classical BIVA)	BIA 101 Anniversary (Akern Srl, Florence, Italy), at 50 kHz	Supine position. Two electrodes were placed on the right wrist and two on the right ankle, with at least 5 cm spacing.	The device was calibrated before assessment using the standard control circuit supplied by the manufacturer with a known impedance (resistance (R) = 380 ohm; reactance (Xc) = 45 ohm). The accuracy of the device was 0.1% for R and 0.1% for Xc.n.s.
**Petri C.**	**2023**	Bioimpedance Patterns and Bioelectrical Impedance Vector Analysis (BIVA) of Body Builders [64]	Italy	Body builders	2 (Classical BIVA)	BIA 101 BIVA PRO (Akern Srl, Florence, Italy), at 50 kHz	Supine position. Two electrodes were placed on the right wrist and two on the right ankle, with at least 5 cm spacing.	It was calibrated every morning using a calibration circuit procedure of known impedance (R = 380 Ohm, Xc = 47 Ohm, 1% error)
**Jiang FL.**	**2023**	Distribution of bioelectrical impedance vector analysis and phase angle in Korean elderly and sarcopenia [70]	Korea	General; Pathological	2 (Classical BIVA); 2 (Classical BIVA)	Quantum Desktop RJL-101 (RJL Systems, Clinton Twp, MI, USA)	Supine position. Two electrodes were placed on the right wrist and two on the right ankle, with at least 5 cm spacing.	Examiner's ICC for repeatability: 0.961 (R), 0.955 (Xc).
**Abdelnour M.**	**2024**	Bioelectrical Impedance Vector Analysis (BIVA) for Assessment of Hydration Status: A Comparison between Endurance and Strength University Athletes [76]	United States	Athletes	4 (Classical BIVA)	InBody 770 (InBody Co.,Ltd., Seoul, Korea) at 50 kHz	Orthostatic position. Hands and feet positioned with heels on round silver electrodes, feet on foot electrodes, thumbs on thumb electrodes, fingers wrapped around bottom electrodes, and arms slightly extended (~15°) from the torso	n.s.
**Cebrián-Ponce Á.**	**2024**	Somatotype and bioelectrical impedance vector analysis of	Italy	Athletes	2 (Classical BIVA); 2	BIA 101 Anniversary (Akern Srl, Florence, Italy), at 50 kHz	Supine position. Two electrodes were placed on the right wrist and two on	The impedance values were R = 383 ± 10 Ω and Xc = 45 ± 5 Ω

		Italian CrossFit® practitioners [77]			(Specific BIVA)		the right ankle, with at least 5 cm spacing.	and a coefficient of variation of 0.2
**Núñez-Ramos R.**	**2024**	Bioelectrical Impedance Vector Analysis in Extremely Low-Birth-Weight Infants to Assess Nutritional Status: Breakthroughs and Insights [78]	Spain	Children-adolescents	3 (Classical BIVA)	BIA 101 BIVA PRO (Akern Srl, Florence, Italy), at 50 kHz	Supine position. Two sets of adhesive Ag/AgCl low-impedance electrodes (BIVATRODES Akern Srl; Florence, Italy) were placed on the hand (blue electrodes) and right foot (gray electrodes) for accurate and sensitive bioimpedance measurements	The device was calibrated every morning using the standard control circuit supplied by the manufacturer with a known impedance (R = 380 ohm; Xc = 45 ohm), and its accuracy was 0.1% for both R and Xc
**Lim S.-K.**	**2025**	The implications of bioelectrical impedance vector analysis in older adults with hip fractures [79]	Korea	Pathological	3 (Classical BIVA)	InBody S10® (InBody Co.,Ltd., Seoul, Korea)	Supine position. Tetra-polar eight-point tactile electrode system	n.s.
**Campa F.**	**2025**	Bioelectrical Impedance Vector Analysis in Older Adults: Reference Standards from a Cross-Sectional Study [80]	Italy	General	2 (Classical BIVA)	BIA-101 (Akern Srl, Florence, Italy) at 50 KHz; BIA 101 Anniversary (Akern Srl, Florence, Italy), at 50 kHz; BIA 101 BIVA PRO (Akern Srl, Florence, Italy), at 50 kHz	Supine position. Two electrodes were placed on the right wrist and two on the right ankle, with at least 5 cm spacing.	Analyzer accuracy was validated using a reference circuit (R: 383 Ω, Xc: 46 Ω). Coefficients of variation were 0.2–0.4% (R) and 0.6–0.8% (Xc); technical error ranged from 2.7–2.9 Ω (R) and 0.4–0.6 Ω (Xc)

Table S4. Overview of included studies and their main characteristics. BIVA, bioelectrical impedance vector analysis; n.s., not specified in the article; R, resistance; Xc, reactance; CV, coefficients of variation.
