# Supplementary material for: Reference Tolerance Ellipses in Bioelectrical Impedance Vector Analysis Across General, Pediatric, Pathological, and Athletic Populations: A Scoping Review
Source: J Funct Morphol Kinesiol. 2025 Oct 22;10(4):415. doi: 10.3390/jfmk10040415 (PMC12641658; doi:10.3390/jfmk10040415)
Supplement: Supplementary file 1 [file jfmk-10-00415-s001.zip › Supplementary Table S5.pdf]

Table S5. General population: characteristics and values for tolerance ellipse construction.

AUTHOR, YEAR	BIVA	SAMPLE SIZE	R/H Mean Ohm/m	R/H SD Ohm/m	XC/H Mean Ohm/m	XC/H SD Ohm/m	*r*	SEX	ETHNICITY	AGE range Years	BMI range Kg/m^2	COUNTRY
**Stolarczyk LM., 1994** [34]	Classical	151	388.5	44.1	50.8	6.9	0.64	F	Mexican–American	18–60	17–38	United States
**Piccoli A., 1995** [35]	Classical	372	371.9	49.0	34.4	7.7	0.41	F	Caucasian	15–85	16–31	Italy
	Classical	354	298.6	43.2	30.8	7.2	0.47	M	Caucasian	15–85	17–31	Italy
**Roubenoff R., 1997** [36]	Classical	167	386.3	38.1	34.1	4.9	0.638	F	Caucasian, Hispanic	75.8 ± 6.6	25.3 ± 4.0	United States
	Classical	116	286.8	31.6	27.4	4.2	0.638	M	Caucasian, Hispanic	76.2 ± 5.6	25.7 ± 3.4	United States
	Classical	294	362.4	45.6	32.8	8.1	0.638	F	Caucasian, Hispanic	78.4 ± 4.5	27.1 ± 4.7	United States
	Classical	161	276	35.4	28.9	9.2	0.638	M	Caucasian, Hispanic	78.2 ± 4.3	28.0 ± 3.7	United States
**Piccoli A., 2002** [37]	Classical	219	409.1	43.9	54.2	7.4	0.68	F	Mexican–American	20–29	19–25	Italy, United States
	Classical	157	378.5	42.6	52.3	7.8	0.74	F	Mexican–American	20–29	25–30	Italy, United States
	Classical	78	347.8	38.4	48.3	6.9	0.66	F	Mexican–American	20–29	30–35	Italy, United States
	Classical	475	411.6	44.4	53.2	7.7	0.66	F	Mexican–American	20–69	19–25	Italy, United States
	Classical	597	373.8	39.5	49.4	7.8	0.60	F	Mexican–American	20–69	25–30	Italy, United States
	Classical	404	346.1	37.2	45.4	7.1	0.60	F	Mexican–American	20–69	30–35	Italy, United States
	Classical	122	405.9	41.0	53.1	7.3	0.72	F	Mexican–American	30–39	19–25	Italy, United States
	Classical	160	365.5	36.9	50.2	6.5	0.64	F	Mexican–American	30–39	25–30	Italy, United States
	Classical	90	345.1	36.3	47.3	6.4	0.68	F	Mexican–American	30–39	30–35	Italy, United States
	Classical	56	421.2	49.9	54.4	7.8	0.78	F	Mexican–American	40–49	19–25	Italy, United States
	Classical	124	373.5	37.9	49.8	7.1	0.62	F	Mexican–American	40–49	25–30	Italy, United States
	Classical	101	345.5	32.9	46.6	6.1	0.62	F	Mexican–American	40–49	30–35	Italy, United States
	Classical	29	411.7	48.4	48.9	7.5	0.72	F	Mexican–American	50–59	19–25	Italy, United States
	Classical	53	382.4	40.5	47.8	7.5	0.67	F	Mexican–American	50–59	25–30	Italy, United States
	Classical	57	344.8	39.0	43.3	6.2	0.64	F	Mexican–American	50–59	30–35	Italy, United States

Classical	49	425.8	43.1	49.9	8.8	0.63	F	Mexican–American	60–69	19–25	Italy, United States
Classical	103	375.8	38.4	44.3	7.9	0.56	F	Mexican–American	60–69	25–30	Italy, United States
Classical	78	347.2	41.3	40.4	6.8	0.67	F	Mexican–American	60–69	30–35	Italy, United States
Classical	288	309.4	35.9	46.6	6.3	0.73	M	Mexican–American	20–29	19–25	Italy, United States
Classical	239	283.6	30.2	43.3	5.6	0.69	M	Mexican–American	20–29	25–30	Italy, United States
Classical	77	257.0	26.5	39.7	5.5	0.76	M	Mexican–American	20–29	30–35	Italy, United States
Classical	554	310.3	34.7	44.7	6.7	0.61	M	Mexican–American	20–69	19–25	Italy, United States
Classical	846	281.9	32.8	40.6	6.1	0.56	M	Mexican–American	20–69	25–30	Italy, United States
Classical	373	254.6	27.1	36.2	6.2	0.64	M	Mexican–American	20–69	30–35	Italy, United States
Classical	119	308.0	31.5	45.0	5.6	0.65	M	Mexican–American	30–39	19–25	Italy, United States
Classical	224	279.1	32.0	41.6	5.5	0.72	M	Mexican–American	30–39	25–30	Italy, United States
Classical	65	254.5	29.0	38.9	5.1	0.78	M	Mexican–American	30–39	30–35	Italy, United States
Classical	53	307.4	28.5	43.2	6.4	0.66	M	Mexican–American	40–49	19–25	Italy, United States
Classical	174	280.0	34.9	40.1	5.8	0.47	M	Mexican–American	40–49	25–30	Italy, United States
Classical	95	251.3	25.0	36.3	5.7	0.79	M	Mexican–American	40–49	30–35	Italy, United States
Classical	29	317.7	30.0	42.2	5.4	0.55	M	Mexican–American	50–59	19–25	Italy, United States
Classical	67	279.8	32.5	38.2	6.2	0.59	M	Mexican–American	50–59	25–30	Italy, United States
Classical	54	252.7	28.8	34.2	5.7	0.70	M	Mexican–American	50–59	30–35	Italy, United States
Classical	65	318.3	40.6	38.5	6.6	0.69	M	Mexican–American	60–69	19–25	Italy, United States
Classical	142	286.8	35.1	36.3	5.7	0.60	M	Mexican–American	60–69	25–30	Italy, United States
Classical	82	257.4	27.6	31.9	5.2	0.66	M	Mexican–American	60–69	30–35	Italy, United States
Classical	207	388.9	48.6	53.9	8.7	0.76	F	Non–hispanic black	20–29	19–25	Italy, United States
Classical	146	349.8	37.5	49.3	7.5	0.75	F	Non–hispanic black	20–29	25–30	Italy, United States
Classical	75	334.3	32.0	48.8	5.7	0.49	F	Non–hispanic black	20–29	30–35	Italy, United States
Classical	528	392.0	45.2	52.8	8.5	0.66	F	Non–hispanic black	20–69	19–25	Italy, United States
Classical	571	354.6	38.4	48.5	7.3	0.67	F	Non–hispanic black	20–69	25–30	Italy, United States
Classical	411	328.6	38.7	45.3	7.6	0.59	F	Non–hispanic black	20–69	30–35	Italy, United States
Classical	167	387.1	37.4	52.6	7.6	0.62	F	Non–hispanic black	30–39	19–25	Italy, United States

Classical	155	355.5	36.6	50.4	7.0	0.73	F	Non–hispanic black	30–39	25–30	Italy, United States
Classical	103	328.6	42.3	47.1	8.0	0.68	F	Non–hispanic black	30–39	30–35	Italy, United States
Classical	75	396.3	44.9	54.2	8.4	0.70	F	Non–hispanic black	40–49	19–25	Italy, United States
Classical	125	349.3	36.3	47.6	6.7	0.68	F	Non–hispanic black	40–49	25–30	Italy, United States
Classical	93	320.8	37.7	45.0	7.0	0.65	F	Non–hispanic black	40–49	30–35	Italy, United States
Classical	35	398.2	46.2	48.8	8.9	0.85	F	Non–hispanic black	50–59	19–25	Italy, United States
Classical	61	358.9	40.0	47.1	7.2	0.75	F	Non–hispanic black	50–59	25–30	Italy, United States
Classical	68	327.4	40.9	42.9	8.0	0.54	F	Non–hispanic black	50–59	30–35	Italy, United States
Classical	44	412.2	50.7	49.8	9.4	0.51	F	Non–hispanic black	60–69	19–25	Italy. United States
Classical	84	365.9	42.9	45.9	7.6	0.65	F	Non–hispanic black	60–69	25–30	Italy, United States
Classical	72	333.8	38.2	41.9	6.7	0.66	F	Non–hispanic black	60–69	30–35	Italy, United States
Classical	217	294.1	33.4	45.4	6.8	0.66	M	Non–hispanic black	20–29	19–25	Italy, United States
Classical	141	264.2	29.7	41.5	6.1	0.74	M	Non–hispanic black	20–29	25–30	Italy, United States
Classical	66	246.4	32.8	39.6	5.8	0.65	M	Non–hispanic black	20–29	30–35	Italy, United States
Classical	643	297.5	37.1	43.0	7.1	0.57	M	Non–hispanic black	20–69	19–25	Italy, United States
Classical	611	267.5	30.8	39.7	6.5	0.65	M	Non–hispanic black	20–69	25–30	Italy, United States
Classical	293	246.4	29.9	36.6	6.4	0.65	M	Non–hispanic black	20–69	30–35	Italy, United States
Classical	182	290.9	35.2	43.5	6.4	0.66	M	Non–hispanic black	30–39	19–25	Italy, United States
Classical	153	264.7	31.1	40.8	6.0	0.72	M	Non–hispanic black	30–39	25–30	Italy, United States
Classical	77	243.1	30.4	38.1	5.7	0.80	M	Non–hispanic black	30–39	30–35	Italy, United States
Classical	115	298.6	35.9	42.0	6.3	0.70	M	Non–hispanic black	40–49	19–25	Italy, United States
Classical	139	268.6	27.3	40.6	6.5	0.67	M	Non–hispanic black	40–49	25–30	Italy, United States
Classical	57	252.3	27.9	38.1	5.7	0.80	M	Non–hispanic black	40–49	30–35	Italy, United States
Classical	55	309.8	43.2	41.4	7.7	0.74	M	Non–hispanic black	50–59	19–25	Italy, United States
Classical	78	267.5	32.2	37.3	5.7	0.72	M	Non–hispanic black	50–59	25–30	Italy, United States
Classical	37	244.3	21.3	32.9	4.8	0.69	M	Non–hispanic black	50–59	30–35	Italy, United States
Classical	74	312.7	43.3	38.0	7.5	0.68	M	Non–hispanic black	60–69	19–25	Italy, United States
Classical	100	275.0	34.5	36.4	7.1	0.73	M	Non–hispanic black	60–69	25–30	Italy, United States

Classical	56	246.2	32.3	32.0	6.0	0.72	M	Non–hispanic black	60–69	30–35	Italy, United States
Classical	280	378.4	44.0	50.2	7.3	0.69	F	Non–hispanic white	20–29	19–25	Italy, United States
Classical	70	353.2	36.7	46.6	7.0	0.65	F	Non–hispanic white	20–29	25–30	Italy, United States
Classical	29	333.8	43.4	44.7	5.4	0.63	F	Non–hispanic white	20–29	30–35	Italy, United States
Classical	1009	383.9	44.8	48.1	7.1	0.59	F	Non–hispanic white	20–69	19–25	Italy, United States
Classical	616	355.0	36.2	44.9	6.6	0.59	F	Non–hispanic white	20–69	25–30	Italy, United States
Classical	350	329.1	36.9	41.9	6.6	0.56	F	Non–hispanic white	20–69	30–35	Italy, United States
Classical	261	370.7	38.5	47.7	6.4	0.69	F	Non–hispanic white	30–39	19–25	Italy, United States
Classical	119	351.0	32.0	46.6	6.7	0.68	F	Non–hispanic white	30–39	25–30	Italy, United States
Classical	73	318.5	30.6	43.1	5.4	0.57	F	Non–hispanic white	30–39	30–35	Italy, United States
Classical	182	386.6	44.0	48.9	7.2	0.73	F	Non–hispanic white	40–49	19–25	Italy, United States
Classical	134	353.1	35.8	45.7	6.3	0.69	F	Non–hispanic white	40–49	25–30	Italy, United States
Classical	77	326.8	33.7	43.6	7.3	0.70	F	Non–hispanic white	40–49	30–35	Italy, United States
Classical	146	393.0	43.6	48.9	6.9	0.64	F	Non–hispanic white	50–59	19–25	Italy, United States
Classical	134	353.1	36.8	43.8	5.6	0.51	F	Non–hispanic white	50–59	25–30	Italy, United States
Classical	84	330.1	31.4	40.7	6.3	0.68	F	Non–hispanic white	50–59	30–35	Italy, United States
Classical	140	406.1	49.3	45.0	6.6	0.57	F	Non–hispanic white	60–69	19–25	Italy, United States
Classical	159	362.1	38.1	42.9	6.9	0.64	F	Non–hispanic white	60–69	25–30	Italy, United States
Classical	87	337.3	44.6	39.6	6.5	0.57	F	Non–hispanic white	60–69	30–35	Italy, United States
Classical	193	286.5	29.5	42.7	5.5	0.58	M	Non–hispanic white	20–29	19–25	Italy, United States
Classical	128	263.9	27.0	40.1	5.6	0.62	M	Non–hispanic white	20–29	25–30	Italy, United States
Classical	36	237.8	23.4	35.8	4.9	0.72	M	Non–hispanic white	20–29	30–35	Italy, United States
Classical	653	292.5	33.8	40.3	6.2	0.56	M	Non–hispanic white	20–69	19–25	Italy, United States
Classical	919	266.4	29.0	36.6	5.8	0.55	M	Non–hispanic white	20–69	25–30	Italy, United States
Classical	369	242.4	25.6	33.1	5.1	0.52	M	Non–hispanic white	20–69	30–35	Italy, United States
Classical	151	290.8	35.5	41.1	6.0	0.75	M	Non–hispanic white	30–39	19–25	Italy, United States
Classical	193	265.3	27.6	38.8	5.1	0.74	M	Non–hispanic white	30–39	25–30	Italy, United States
Classical	62	239.7	27.5	34.7	4.4	0.68	M	Non–hispanic white	30–39	30–35	Italy, United States

	Classical	115	291.1	32.4	39.9	5.8	0.69	M	Non–hispanic white	40–49	19–25	Italy, United States
	Classical	188	263.3	27.9	37.4	5.3	0.61	M	Non–hispanic white	40–49	25–30	Italy, United States
	Classical	71	238.7	25.6	33.9	5.3	0.66	M	Non–hispanic white	40–49	30–35	Italy, United States
	Classical	97	296.4	37.4	37.9	6.3	0.67	M	Non–hispanic white	50–59	19–25	Italy, United States
	Classical	180	267.2	28.6	35.4	4.8	0.66	M	Non–hispanic white	50–59	25–30	Italy, United States
	Classical	89	241.6	22.4	32.3	4.7	0.41	M	Non–hispanic white	50–59	30–35	Italy, United States
	Classical	97	304.5	33.8	37.2	6.0	0.60	M	Non–hispanic white	60–69	19–25	Italy, United States
	Classical	230	270.4	32.5	33.1	5.3	0.64	M	Non–hispanic white	60–69	25–30	Italy, United States
	Classical	111	248.3	26.8	31.5	5.1	0.60	M	Non–hispanic white	60–69	30–35	Italy, United States
**Bogonez P., 2003** [29]	Classical	33	264.3	31.7	30.4	3.5	0.44	M	Caucasian	50	19–30	Spain
**Nescolarde L., 2004** [50]	Classical	507	394.1	53.2	41.6	7.3	0.67	F	n.s.	18–70	19–30	Cuba
	Classical	689	299.4	38.3	33.7	5.3	0.57	M	n.s.	18–70	19–30	Cuba
**Bosy–Westphal A., 2005** [44]	Classical	1052	444.6	69.4	45.71	5.65	0.69	F	n.s.	18–19	18.5–25	Germany
	Classical	1129	464.5	76.3	48.92	5.99	0.73	F	n.s.	18–19	25–30	Germany
	Classical	582	491.1	74.4	51.95	6.34	0.72	F	n.s.	18–19	30–35	Germany
	Classical	239	530.9	100.6	56.11	7.66	0.75	F	n.s.	18–19	35–40	Germany
	Classical	95	572.8	97.9	60.15	8.04	0.80	F	n.s.	18–19	40–50	Germany
	Classical	8307	447	71.8	46.26	5.64	0.71	F	n.s.	20–29	18.5–25	Germany
	Classical	11117	462.7	73.7	48.92	5.98	0.72	F	n.s.	20–29	25–30	Germany
	Classical	6507	485.4	80.2	51.99	6.61	0.73	F	n.s.	20–29	30–35	Germany
	Classical	2857	517	86.1	55.66	7.39	0.75	F	n.s.	20–29	35–40	Germany
	Classical	1306	561.4	94.7	59.84	7.89	0.74	F	n.s.	20–29	40–50	Germany
	Classical	10162	446.9	70.5	46.71	5.77	0.72	F	n.s.	30–39	18.5–25	Germany
	Classical	18824	461.8	73.4	49.43	6.04	0.73	F	n.s.	30–39	25–30	Germany
	Classical	11506	486.8	77.7	52.78	6.55	0.74	F	n.s.	30–39	30–35	Germany
	Classical	5064	523.6	87.5	56.63	7.21	0.75	F	n.s.	30–39	35–40	Germany

Classical	2537	572.5	98.3	61.33	8.16	0.75	F	n.s.	30–39	40–50	Germany
Classical	6691	447.8	72.8	46.21	5.76	0.72	F	n.s.	40–49	18.5–25	Germany
Classical	17090	463.2	73.5	48.91	5.93	0.72	F	n.s.	40–49	25–30	Germany
Classical	12495	491	79.7	52.47	6.6	0.72	F	n.s.	40–49	30–35	Germany
Classical	5462	528.4	88.9	56.33	7.17	0.73	F	n.s.	40–49	35–40	Germany
Classical	2709	586.5	110.1	61.61	8.79	0.76	F	n.s.	40–49	40–50	Germany
Classical	3408	462.6	76.1	45.87	5.59	0.69	F	n.s.	50–59	18.5–25	Germany
Classical	13137	479.2	78.5	48.65	5.96	0.7	F	n.s.	50–59	25–30	Germany
Classical	11817	509.7	86	52.08	6.59	0.71	F	n.s.	50–59	30–35	Germany
Classical	5178	547.7	97.8	55.69	7.43	0.72	F	n.s.	50–59	35–40	Germany
Classical	2280	603.1	117.4	60.42	8.56	0.77	F	n.s.	50–59	40–50	Germany
Classical	1106	478.1	85.4	45.35	5.9	0.64	F	n.s.	60–69	18.5–25	Germany
Classical	5649	498.4	84.9	48.1	5.98	0.67	F	n.s.	60–69	25–30	Germany
Classical	6305	527.4	94.1	51.09	6.61	0.68	F	n.s.	60–69	30–35	Germany
Classical	3090	570.6	108.7	54.6	7.38	0.7	F	n.s.	60–69	35–40	Germany
Classical	1274	631.4	128.6	59.6	8.65	0.73	F	n.s.	60–69	40–50	Germany
Classical	276	508.1	105.1	44.46	6.5	0.59	F	n.s.	≥70	18.5–25	Germany
Classical	1124	524.3	102.1	47.28	6.5	0.69	F	n.s.	≥70	25–30	Germany
Classical	1419	557.3	111.4	50.47	7.2	0.73	F	n.s.	≥70	30–35	Germany
Classical	685	598.9	120	53.86	7.44	0.66	F	n.s.	≥70	35–40	Germany
Classical	243	659.6	142.4	57.21	7.92	0.7	F	n.s.	≥70	40–50	Germany
Classical	115	555.8	95.1	5.64	8.46	0.70	M	n.s.	18–19	18.5–25	Germany
Classical	138	564.4	88.8	69.38	9.39	0.78	M	n.s.	18–19	25–30	Germany
Classical	115	629.3	135.0	74.89	9.52	0.41	M	n.s.	18–19	30–35	Germany
Classical	55	659.5	86.6	78.46	10.24	0.73	M	n.s.	18–19	35–40	Germany
Classical	30	702.9	102.6	80.11	9.00	0.77	M	n.s.	18–19	40–50	Germany
Classical	614	547.7	91.3	65.51	9.04	0.75	M	n.s.	20–29	18.5–25	Germany
Classical	1360	572.8	94.0	69.70	9.09	0.76	M	n.s.	20–29	25–30	Germany

Classical	1200	603.2	99.7	73.52	9.55	0.76	M	n.s.	20–29	30–35	Germany
Classical	518	651.4	105.6	78.04	10.00	0.72	M	n.s.	20–29	35–40	Germany
Classical	196	719.9	123.0	84.08	11.17	0.79	M	n.s.	20–29	40–50	Germany
Classical	639	560.9	100.1	64.74	8.55	0.73	M	n.s.	30–39	18.5–25	Germany
Classical	2747	579.9	92.5	69.65	8.56	0.59	M	n.s.	30–39	25–30	Germany
Classical	2682	621.6	103.9	74.86	9.48	0.75	M	n.s.	30–39	30–35	Germany
Classical	1090	668.9	107.6	80.01	10.84	0.76	M	n.s.	30–39	35–40	Germany
Classical	444	754.5	145.0	87.10	12.44	0.78	M	n.s.	30–39	40–50	Germany
Classical	464	561.1	91.6	62.92	8.13	0.72	M	n.s.	40–49	18.5–25	Germany
Classical	2494	591.9	100.7	68.77	8.93	0.74	M	n.s.	40–49	25–30	Germany
Classical	2809	633.2	105.7	74.12	9.35	0.75	M	n.s.	40–49	30–35	Germany
Classical	1187	692.2	124.7	79.52	10.83	0.77	M	n.s.	40–49	35–40	Germany
Classical	487	781.4	149.0	86.74	12.56	0.76	M	n.s.	40–49	40–50	Germany
Classical	294	578.6	103.4	62.54	8.74	0.80	M	n.s.	50–59	18.5–25	Germany
Classical	1994	610.0	106.3	67.66	8.48	0.71	M	n.s.	50–59	25–30	Germany
Classical	2542	656.1	111.7	73.02	9.17	0.74	M	n.s.	50–59	30–35	Germany
Classical	1060	708.6	137.3	77.80	10.46	0.71	M	n.s.	50–59	35–40	Germany
Classical	417	804.2	166.9	85.34	12.55	0.77	M	n.s.	50–59	40–50	Germany
Classical	218	621.6	119.6	61.52	7.40	0.59	M	n.s.	60–69	18.5–25	Germany
Classical	1267	637.3	116.8	66.15	8.78	0.71	M	n.s.	60–69	25–30	Germany
Classical	1643	683.5	129.1	70.99	9.26	0.71	M	n.s.	60–69	30–35	Germany
Classical	643	740.2	152.0	75.67	10.35	0.72	M	n.s.	60–69	35–40	Germany
Classical	212	823.0	186.3	81.75	11.66	0.67	M	n.s.	60–69	40–50	Germany
Classical	86	666.2	132.8	58.13	7.26	0.47	M	n.s.	≥70	18.5–25	Germany
Classical	313	696.1	138.9	64.83	8.60	0.68	M	n.s.	≥70	25–30	Germany
Classical	381	719.4	141.6	67.97	8.98	0.70	M	n.s.	≥70	30–35	Germany
Classical	89	790.7	147.7	73.90	10.53	0.66	M	n.s.	≥70	35–40	Germany
Classical	29	975.8	276.8	82.95	11.98	0.81	M	n.s.	≥70	40–50	Germany

**Lukaski H.C., 2007** [51]	Classical	15[a]	361	10	44	1	0.61	F	n.s.	21–37	18–30	United States
	Classical	15[b]	355	10	41	1	0.65	F	n.s.	21–37	18–30	United States
	Classical	15[c]	347	10	40	1	0.66	F	n.s.	21–37	18–30	United States
	Classical	13[d]	365	10	42	1	0.72	F	n.s.	21–37	18–30	United States
	Classical	14[e]	318	10	36	1	0.86	F	n.s.	21–37	18–30	United States
**Buffa R., 2013** [25]	Classical	836	264.4	37.1	34.4	5.2	0.741	M	Multiethnic	34.2 ± 8.6	27.3 ± 4.8	United States
**Siváková D., 2013** [52]	Classical	202	378.18	40.35	42.93	5.78	0.67	F	n.s.	18–28	n.s.	Slovakia
	Classical	55	352.02	31.29	42.51	6.20	0.73	F	n.s.	29–39	n.s.	Slovakia
	Classical	256	336.37	42.41	38.17	6.60	0.63	F	n.s.	40–49	n.s.	Slovakia
	Classical	215	335.38	43.33	37.56	6.12	0.65	F	n.s.	50–59	n.s.	Slovakia
	Classical	102	330.66	50.46	33.10	7.40	0.44	F	n.s.	60–69	n.s.	Slovakia
	Classical	125	339.71	51.36	31.55	8.57	0.54	F	n.s.	70–79	n.s.	Slovakia
	Classical	52	349.52	47.15	32.57	12.16	0.51	F	n.s.	80–92	n.s.	Slovakia
	Classical	193	263.76	30.00	35.58	6.40	0.53	M	n.s.	18–28	n.s.	Slovakia
	Classical	45	255.38	34.30	35.82	5.48	0.73	M	n.s.	29–39	n.s.	Slovakia
	Classical	22	251.42	30.73	31.00	3.98	0.86	M	n.s.	40–49	n.s.	Slovakia
	Classical	32	252.60	24.75	30.92	6.27	0.47	M	n.s.	50–59	n.s.	Slovakia
	Classical	104	270.68	42.47	29.16	8.37	0.59	M	n.s.	60–69	n.s.	Slovakia
	Classical	110	279.48	51.13	26.66	7.23	0.71	M	n.s.	70–79	n.s.	Slovakia
	Classical	30	285.62	56.46	25.12	8.60	0.65	M	n.s.	80–92	n.s.	Slovakia
**Nescolarde L., 2013** [49]	Classical	1399	398.0	46.8	42.0	5.2	0.71	F	Multiethnic	17–59	22.8 ± 2.2	Cuba
	Classical	128	404.4	59.6	38.0	5.4	0.72	F	Multiethnic	60–80	23.6 ± 2.4	Cuba

	Classical	1263	292.3	32.4	33.8	3.6	0.72	M	Multiethnic	17–59	23.5 ± 2.2	Cuba
	Classical	174	318.2	42.2	32.0	4.1	0.64	M	Multiethnic	60–80	23.1 ± 2.1	Cuba
**Saragat B., 2014** [27]	Classical	265	300.6	44.9	32.4	6.2	0.43	M	Caucasian	77.0 ± 7.2	26.4 ± 3.3	Italy
	Classical	295	370.4	47.7	38.1	6.3	0.44	F	Caucasian	76.0 ± 7.1	26.6 ± 4.1	Italy
**Jensen B., 2019** [66]	Classical	192	411	35	36.4	4.2	0.67	F	Caucasian	<40	≥18.5≤25	German
	Classical	50	383	34	35.2	4.3	0.69	F	Caucasian	<40	≥25≤30	German
	Classical	133	401	37	34.5	4.7	0.73	F	Caucasian	>40	≥18.5≤25	German
	Classical	82	376	33	33.3	4.0	0.60	F	Caucasian	>41	≥25≤30	German
	Classical	38	349	32	30.4	3.9	0.67	F	Caucasian	>42	≥30	German
	Classical	361	407	36	35.6	4.5	0.70	F	Caucasian	18–65	≥18.5≤25	German
	Classical	289	379	33	34.0	4.2	0.64	F	Caucasian	18–65	≥25≤30	German
	Classical	58	351	32	31.5	4.2	0.68	F	Caucasian	18–65	≥30	German
	Classical	139	327	30	34.4	4.2	0.78	M	Caucasian	<40	≥18.5≤25	German
	Classical	111	298	24	32.3	3.8	0.72	M	Caucasian	<40	≥25≤30	German
	Classical	66	311	27	30.7	4.0	0.75	M	Caucasian	>40	≥18.5≤25	German
	Classical	153	292	26	29.1	3.7	0.76	M	Caucasian	>40	≥25≤30	German
	Classical	47	265	23	27.3	3.4	0.76	M	Caucasian	>40	≥30	German
	Classical	205	322	30	33.2	4.5	0.78	M	Caucasian	18–65	≥18.5≤25	German
	Classical	264	295	25	30.4	4.1	0.73	M	Caucasian	18–65	≥25≤30	German
	Classical	62	270	25	27.7	3.4	0.77	M	Caucasian	18–65	≥30	German
	Classical	41	497	42	39.6	5.0	0.77	F	Asiatic	<40	<18.5	Japan
	Classical	190	447	43	36.8	4.3	0.69	F	Asiatic	<40	≥18.5≤25	Japan
	Classical	34	472	31	36.2	4.8	0.49	F	Asiatic	>40	<18.5	Japan
	Classical	191	427	39	34.2	4.5	0.72	F	Asiatic	>40	≥18.5≤25	Japan
	Classical	75	486	40	38.1	5.2	0.70	F	Asiatic	21–87	<18.5	Japan
	Classical	381	437	42	35.5	4.6	0.72	F	Asiatic	21–87	≥18.5≤25	Japan
	Classical	33	407	44	35.5	4.8	0.79	F	Asiatic	21–87	≥25≤30	Japan

	Classical	189	347	32	34.1	4.1	0.62	M	Asiatic	<40	≥18.5≤25	Japan
	Classical	32	311	18	31.3	2.8	0.64	M	Asiatic	<40	≥25≤30	Japan
	Classical	159	337	34	30.4	4.3	0.69	M	Asiatic	>40	≥18.5≤25	Japan
	Classical	69	312	24	30.2	4.1	0.45	M	Asiatic	>40	≥25≤30	Japan
	Classical	25	388	41	34.1	5.4	0.82	M	Asiatic	21–87	<18.5	Japan
	Classical	348	342	33	32.4	4.6	0.65	M	Asiatic	21–87	≥18.5≤25	Japan
	Classical	101	312	22	30.6	3.7	0.48	M	Asiatic	21–87	≥25≤30	Japan
	Classical	130	476	43	42.5	4.3	0.60	F	Mexican	<40	≥18.5≤25	Mexico
	Classical	77	432	43	41.1	5.1	0.77	F	Mexican	<40	≥25≤30	Mexico
	Classical	44	391	44	37	5.1	0.60	F	Mexican	<40	≥30	Mexico
	Classical	65	464	40	39.6	4.6	0.74	F	Mexican	>40	≥18.5≤25	Mexico
	Classical	112	432	35	39.4	4.6	0.64	F	Mexican	>40	≥25≤30	Mexico
	Classical	74	397	39	37	4.4	0.69	F	Mexican	>40	≥30	Mexico
	Classical	336	472	42	41.6	4.6	0.65	F	Mexican	18–67	≥18.5≤25	Mexico
	Classical	526	432	38	40.1	4.9	0.69	F	Mexican	18–67	≥25≤30	Mexico
	Classical	417	395	41	37	4.6	0.65	F	Mexican	18–67	≥30	Mexico
	Classical	84	367	29	38.8	4.3	0.83	M	Mexican	<40	≥18.5≤25	Mexico
	Classical	108	338	33	36.9	4.6	0.77	M	Mexican	<40	≥25≤30	Mexico
	Classical	70	303	27	33.2	4.6	0.77	M	Mexican	<40	≥30	Mexico
	Classical	38	357	24	35.6	3.9	0.74	M	Mexican	>40	≥18.5≤25	Mexico
	Classical	144	331	27	34.3	3.7	0.74	M	Mexican	>40	≥25≤30	Mexico
	Classical	76	302	30	31.9	4.3	0.70	M	Mexican	>40	≥30	Mexico
	Classical	414	364	28	37.8	4.4	0.81	M	Mexican	18–67	≥18.5≤25	Mexico
	Classical	180	334	30	35.4	4.3	0.75	M	Mexican	18–67	≥25≤30	Mexico
	Classical	102	303	29	32.5	4.5	0.73	M	Mexican	18–67	≥30	Mexico
**Oh J.–H., 2019** [67]	Classical	105	425.6	46.1	39.7	4.8	0.720	F	Asiatic	37.9 ± 14.9	22.0 ± 3.2	Korea
	Classical	137	306.3	34.6	34.9	4.3	0.746	M	Asiatic	37.3 ± 11.5	25.1 ± 3.5	Korea

**Jiang FL., 2023** [70]	Classical	57	353.0	34.2	31.9	3.7	0.54	F	Asiatic	75.1 ± 4.4	22.9 ± 2.2	Korea
	Classical	65	266.9	22.4	28.2	3.4	0.63	M	Asiatic	76.6 ± 4.2	23.6 ± 2.3	Korea
**Rossini–Venturini A.C., 2022** [68]	Classical	25	281.1	38.2	29.0	7.7	0.075	M	n.s.	71.0 ± 7.6	26.2 ± 3.5	Brazil
	Classical	59	346.0	42.0	36.4	6.7	0.447	F	n.s.	69.5 ± 5.8	27.7 ± 4.3	Brazil
**Campa F., 2023** [69]	Classical	2230	337.2	47.8	35.9	5.5	0.67	F	Caucasian	18–65	23.9 ± 3.2	Italy
	Classical	2137	265.7	35.1	32.1	4.9	0.60	M	Caucasian	18–65	25.1 ± 3.1	Italy
**Campa F., 2025** [80]	Classical	472	363.80	63.90	30.60	6.10	0.70	F	Caucasian	73.90 ± 7.40	27.20 ± 5.40	Italy
	Classical	363	280.20	47.90	26.80	4.10	0.60	M	Caucasian	73.10 ± 7.20	27.00 ± 4.40	Italy
**Buffa R., 2013** [25]	Specific	836	402.4	62.9	52.5	9.5	0.839	M	Multiethnic	34.2 ± 8.6	27.3 ± 4.8	United States
	Specific	754	344.3	49.6	38.6	5.9	0.741	F	Multiethnic	35.5 ± 8.4	28.3 ± 7.0	United States
	Specific	754	492.0	95.9	55.4	12.3	0.875	F	Multiethnic	35.5 ± 8.4	28.3 ± 7.0	United States
**Saragat B., 2014** [27]	Specific	265	391.8	57.9	42.6	9.9	0.59	M	Caucasian	77.0 ± 7.2	26.4 ± 3.3	Italy
	Specific	295	462.0	80.1	47.9	11.2	0.75	F	Caucasian	76.0 ± 7.1	26.6 ± 4.1	Italy
**Ibáñez M.E., 2015** [65]	Specific	227	388.6	60.0	43.7	7.5	0.79	F	n.s.	22.5 ± 2.8	22.3 ± 3.3	Italy, Spain
	Specific	213	332.7	41.6	44.4	6.8	0.77	M	n.s.	22.3 ± 2.6	23.4 ± 2.9	Italy, Spain
**Rossini–Venturini A.C., 2022** [68]	Specific	25	372.6	43.5	39.1	12.1	0.489	M	n.s.	71.0 ± 7.6	26.2 ± 3.5	Brazil
	Specific	59	433.4	71.3	45.7	9.8	0.659	F	n.s.	69.5 ± 5.8	27.7 ± 4.3	Brazil

Table S5. General population: characteristics and values for tolerance ellipse construction. BIVA, bioelectrical impedance vector analysis; R/H, resistance-to-height ratio; Xc/H, reactance-to-height ratio; SD, standard deviation; BMI, body mass index; M, male; F, female; n.s., not specified in the article; a, before pregnancy; b postpartum; c second-trimester pregnancy; d first-trimester pregnancy; e third-trimester pregnancy.
