# Supplementary material for: Reference Tolerance Ellipses in Bioelectrical Impedance Vector Analysis Across General, Pediatric, Pathological, and Athletic Populations: A Scoping Review
Source: J Funct Morphol Kinesiol. 2025 Oct 22;10(4):415. doi: 10.3390/jfmk10040415 (PMC12641658; doi:10.3390/jfmk10040415)
Supplement: Supplementary file 1 [file jfmk-10-00415-s001.zip › Supplementary Table S6.pdf]

Table S6. Children-adolescents population: characteristics and values for tolerance ellipse construction.

AUTHOR, YEAR	BIVA	CATEGORY	SAMPLE SIZE	R/H Mean Ohm/m	R/H SD Ohm/m	XC/H Mean Ohm/m	XC/H SD Ohm/m	*r*	SEX	ETHNICITY	AGE range Years	BMI range Kg/m^2	COUNTRY
**De Palo T., 2000** [26]	Classical	Infancy	115	751	75	60	10	0.41	F/M	Caucasian	2–3	16.3 ± 1.1	Italy
	Classical	Childhood	220	672	72	58	8	0.57	F/M	Caucasian	4–5	15.6 ± 1.1	Italy
	Classical	Childhood	448	600	64	56	7	0.56	F/M	Caucasian	6–7	15.6 ± 1.2	Italy
	Classical	Childhood	227	561	58	54	7	0.57	F/M	Caucasian	8	16.1 ± 1.4	Italy
	Classical	Childhood	200	527	53	52	7	0.66	F/M	Caucasian	9	16.5 ± 1.4	Italy
	Classical	Childhood	369	486	58	48	6	0.71	F/M	Caucasian	10–11	17.1 ± 1.6	Italy
	Classical	Adolescence	132	440	57	44	6	0.77	F/M	Caucasian	12	18.1 ± 2.0	Italy
	Classical	Adolescence	116	402	61	42	6	0.74	F/M	Caucasian	13	19.3 ± 2.1	Italy
	Classical	Adolescence	120	403	44	41	6	0.67	F	Caucasian	14–15	20.9 ± 2.2	Italy
	Classical	Adolescence	97	338	48	37	5	0.67	M	Caucasian	14–15	20.0 ± 2.0	Italy
**Piccoli A., 2002** [42]	Classical	Infancy	36	1042	119	79	20	0.36	F/M	Caucasian	1 days	12.7 ± 1.0	Italy
	Classical	Infancy	65	994	122	89	29	0.25	F/M	Caucasian	2 days	12.6 ± 0.9	Italy
	Classical	Infancy	163	1002	128	85	27	0.31	F/M	Caucasian	1–7 days	12.6 ± 1.0	Italy
	Classical	Infancy	62	987	137	84	26	0.42	F/M	Caucasian	3–7 days	12.6 ± 1.2	Italy
	Classical	Infancy	63	801	145	47	22	0.28	F	Caucasian	0–11.99 months	15.4 ± 1.7	Italy
	Classical	Infancy	153	757	137	41	19	0.13	F/M	Caucasian	0–11.99 months	15.5 ± 1.7	Italy
	Classical	Infancy	90	726	121	37	16	-0.17	M	Caucasian	0–11.99 months	15.6 ± 1.7	Italy
	Classical	Infancy	20	861	136	51	28	0.56	F	Caucasian	0–3.99 months	14.6 ± 2.1	Italy
	Classical	Infancy	58	808	120	39	22	0.26	F/M	Caucasian	0–3.99 months	14.8 ± 1.6	Italy
	Classical	Infancy	38	780	102	32	14	-0.42	M	Caucasian	0–3.99 months	14.8 ± 1.3	Italy
	Classical	Infancy	21	815	171	44	15	0.23	F	Caucasian	4–7.99 months	15.5 ± 1.3	Italy
	Classical	Infancy	51	761	145	41	18	0.28	F/M	Caucasian	4–7.99 months	15.7 ± 1.6	Italy
	Classical	Infancy	30	724	112	39	19	0.30	M	Caucasian	4–7.99 months	15.9 ± 1.7	Italy

	Classical	Infancy	22	733	98	47	20	-0.10	F	Caucasian	8–11.99 months	16.0 ± 1.3	Italy
	Classical	Infancy	44	684	116	45	17	-0.04	F/M	Caucasian	8–11.99 months	16.2 ± 1.6	Italy
	Classical	Infancy	22	635	113	44	12	-0.06	M	Caucasian	8–11.99 months	16.4 ± 1.9	Italy
**Bosy-Westphal A., 2005** [44]	Classical	Childhood	72	191.5	52.6	16.86	4.61	0.77	F	n.s.	6–9	9–13	Germany
	Classical	Childhood	853	197.2	34.9	17.44	2.80	0.71	F	n.s.	6–9	>13–15	Germany
	Classical	Childhood	879	259.4	75.6	24.72	8.02	0.91	F	n.s.	6–9	>17–25	Germany
	Classical	Childhood	1168	209.5	40.3	19.19	3.60	0.65	F	n.s.	6–9	>15–17	Germany
	Classical	Childhood	39	204.1	39.7	16.85	2.51	0.74	M	n.s.	6–9	9–13	Germany
	Classical	Childhood	670	263.1	62.1	25.04	6.55	0.84	M	n.s.	6–9	>17–25	Germany
	Classical	Childhood	860	214.4	71.3	18.59	2.68	0.33	M	n.s.	6–9	>13–15	Germany
	Classical	Childhood	1194	225.3	44.5	20.62	9.35	0.27	M	n.s.	6–9	>15–17	Germany
	Classical	Adolescence	190	272.7	55.2	24.54	3.57	0.74	F	n.s.	10–13	11-15	Germany
	Classical	Adolescence	293	490.2	86.6	49.75	7.55	0.73	F	n.s.	10–13	>30–35	Germany
	Classical	Adolescence	654	439.7	80.4	43.92	7.82	0.80	F	n.s.	10–13	>25–30	Germany
	Classical	Adolescence	748	376.1	80.7	36.64	7.92	0.84	F	n.s.	10–13	>20–25	Germany
	Classical	Adolescence	1113	304.5	61.5	28.86	5.50	0.77	F	n.s.	10–13	>15–20	Germany
	Classical	Adolescence	165	508.3	95.0	51.27	10.93	0.81	M	n.s.	10–13	>30–35	Germany
	Classical	Adolescence	186	285.1	51.0	25.93	3.84	0.78	M	n.s.	10–13	11-15	Germany
	Classical	Adolescence	379	433.9	82.6	43.61	8.74	0.84	M	n.s.	10–13	>25–30	Germany
	Classical	Adolescence	484	372.2	84.9	36.60	8.91	0.81	M	n.s.	10–13	>20–25	Germany
	Classical	Adolescence	1125	313.9	58.0	29.91	4.86	0.77	M	n.s.	10–13	>15–20	Germany
	Classical	Adolescence	157	420.9	75.3	40.78	5.30	0.71	F	n.s.	14–17	14-19	Germany
	Classical	Adolescence	271	529.9	92.2	55.90	7.94	0.75	F	n.s.	14–17	>35–40	Germany
	Classical	Adolescence	796	489.9	79.5	52.10	6.55	0.73	F	n.s.	14–17	>30–35	Germany
	Classical	Adolescence	1138	441.3	69.8	45.40	5.79	0.72	F	n.s.	14–17	>19–25	Germany
	Classical	Adolescence	1375	462.1	73.6	48.61	6.15	0.71	F	n.s.	14–17	>25–30	Germany
	Classical	Adolescence	34	494.1	100.1	49.31	9.97	0.85	M	n.s.	14–17	14-19	Germany
	Classical	Adolescence	94	656.0	107.3	72.16	11.28	0.55	M	n.s.	14–17	>35–40	Germany

	Classical	Adolescence	167	535.7	100.7	59.31	10.01	0.73	M	n.s.	14–17	>19–25	Germany
	Classical	Adolescence	249	605.0	99.2	66.02	11.68	0.74	M	n.s.	14–17	>30–35	Germany
	Classical	Adolescence	252	558.8	96.1	60.82	12.06	0.75	M	n.s.	14–17	>25–30	Germany
**Guida B., 2008** [45]	Classical	Childhood	111	476.8	61.4	52.8	11.5	0.34	F/M	Caucasian	8	24.1 ± 2.3	Italy
	Classical	Childhood	135	520.7	52.9	55.1	11.0	0.21	F/M	Caucasian	8	19.9 ± 0.9	Italy
	Classical	Childhood	218	567.9	68.4	59.0	11.2	0.36	F/M	Caucasian	8	16.4 ± 1.2	Italy
	Classical	Childhood	464	532.4	72.6	56.4	11.5	0.25	F/M	Caucasian	8	19.3 ± 3.4	Italy
**Margutti A.V.B., 2010** [46]	Classical	Infancy	52	1324.1	87.9	72.0	9.8	0.37	M	Multiethnic	7–28 days	14.0 ± 1.0	Brazil
	Classical	Infancy	57	1377.3	138.0	75.8	11.4	0.36	F	Multiethnic	7–28 days	13.5 ± 1.3	Brazil
	Classical	Infancy	109	1351.9	119.3	74.0	10.8	0.38	F/M	Multiethnic	7–28 days	13.8 ± 1.2	Brazil
**L’Abée C., 2010** [47]	Classical	Infancy	113	789	97	63	21	0.29	F/M	n.s.	2 months	15.6 ± 1.7	Netherlands
	Classical	Infancy	53	723	117	68	23	0.09	F/M	n.s.	6–7 months	16.8 ± 1.3	Netherlands
	Classical	Infancy	46	692	145	73	21	0.05	F/M	n.s.	8–12 months	17.0 ± 1.3	Netherlands
**Tanabe R.F., 2012** [48]	Classical	Infancy	103	817.6	89.0	46.0	8.1	0.45	F/M	Multiethnic	1–6 months	16.7 ± 1.4	Brazil
	Classical	Infancy	126	794.9	92.2	50.2	8.1	0.30	F	Multiethnic	1–36 months	16.5 ± 1.5	Brazil
	Classical	Infancy	129	745.5	86.9	45.5	6.4	0.10	M	Multiethnic	1–36 months	17.0 ± 1.4	Brazil
	Classical	Infancy	55	764.3	80.8	47.4	6.2	0.39	F/M	Multiethnic	6–12 months	17.5 ± 1.4	Brazil
	Classical	Infancy	97	721.0	76.0	50.2	7.2	0.47	F/M	Multiethnic	12–36 months	16.4 ± 1.3	Brazil
**Nescolarde L., 2013** [49]	Classical	Infancy	61	741.4	79.2	70.9	8.1	0.67	F/M	Multiethnic	2–3	16.2 ± 0.9	Cuba
	Classical	Childhood	91	651.2	65.6	63.2	6.4	0.55	F/M	Multiethnic	4–5	15.9 ± 1.0	Cuba
	Classical	Childhood	165	578.5	52.4	57.5	6.3	0.60	F/M	Multiethnic	6–7	15.9 ± 1.0	Cuba
	Classical	Childhood	179	513.0	51.1	51.0	5.2	0.67	F/M	Multiethnic	8–9	16.6 ± 1.3	Cuba
	Classical	Childhood	196	461.3	59.6	47.0	5.9	0.59	F/M	Multiethnic	10–11	17.6 ± 1.8	Cuba
	Classical	Adolescence	109	419.6	50.8	43.6	5.0	0.71	F/M	Multiethnic	12	19.0 ± 1.9	Cuba
	Classical	Adolescence	161	413.0	45.8	43.6	4.7	0.69	F	Multiethnic	13–16	20.4 ± 2.0	Cuba
	Classical	Adolescence	101	335.2	48.7	37.0	4.9	0.71	M	Multiethnic	13–16	19.5 ± 2.0	Cuba
**Mathias-Genovez M.G., 2015** [71]	Classical	Childhood	26	482.5	65.2	44.6	5.4	0.73	F	n.s.	10	17.1 ± 1.5	Brazil

	Classical	Childhood	21	472.8	57.5	47.4	5.1	0.31	M	n.s.	10	16.6 ± 1.4	Brazil
	Classical	Childhood	44	451.3	49.7	42.7	5.3	0.44	F	n.s.	11	17.1 ± 1.7	Brazil
	Classical	Childhood	36	437.4	49.2	45.8	5.1	0.57	M	n.s.	11	17.1 ± 1.4	Brazil
	Classical	Adolescence	47	418.0	47.9	41.3	4.2	0.44	F	n.s.	12	18.1 ± 1.7	Brazil
	Classical	Adolescence	35	395.0	68.1	41.1	7.2	0.85	M	n.s.	12	17.6 ± 1.7	Brazil
	Classical	Adolescence	52	410.9	49.8	41.4	5.0	0.67	F	n.s.	13	19.0 ± 1.5	Brazil
	Classical	Adolescence	30	356.9	45.1	37.8	4.1	0.61	M	n.s.	13	17.9 ± 1.4	Brazil
	Classical	Adolescence	46	410.7	34.3	43.3	6.3	0.43	F	n.s.	14	19.2 ± 1.5	Brazil
	Classical	Adolescence	44	311.6	46.6	36.6	4.1	0.69	M	n.s.	14	19.4 ± 1.5	Brazil
	Classical	Adolescence	38	393.3	39.5	42.2	4.1	0.42	F	n.s.	15	20.3 ± 1.9	Brazil
	Classical	Adolescence	34	306.0	28.8	37.8	4.1	0.03	M	n.s.	15	19.5 ± 1.8	Brazil
	Classical	Adolescence	30	384.6	34.1	41.5	5.1	0.46	F	n.s.	16	20.5 ± 1.8	Brazil
	Classical	Adolescence	32	284.9	33.7	35.9	4.7	0.42	M	n.s.	16	20.7 ± 1.8	Brazil
	Classical	Adolescence	18	399.5	46.7	43.7	4.1	0.46	F	n.s.	17	20.8 ± 1.7	Brazil
	Classical	Adolescence	13	284.3	32.8	37.1	3.1	0.83	M	n.s.	17	20.6 ± 1.6	Brazil
	Classical	Adolescence	11	395.8	41.5	41.4	4.5	0.72	F	n.s.	18	21.2 ± 2.1	Brazil
	Classical	Adolescence	10	296.5	29.8	37.2	3.8	0.61	M	n.s.	18	19.8 ± 2.0	Brazil
**Toffano R.B.D., 2017** [72]	Classical	Infancy	73	911.3	99.6	67.6	9.3	0.53	M	Multiethnic	30–90 days	16.1 ± 1.4	Brazil
	Classical	Infancy	77	942.1	97.0	71.5	8.7	0.68	F	Multiethnic	30–90 days	15.39 ± 1.5	Brazil
	Classical	Infancy	150	927.1	99.1	69.6	9.2	0.38	F/M	Multiethnic	30–90 days	15.7 ± 1.5	Brazil
**Redondo-del-Río M.P., 2017** [73]	Classical	Adolescence	558	310.3	34.4	37.8	4.7	0.62	M	n.s.	16≤19	22.6 ± 2.9	Spain
	Classical	Childhood	87	736.1	76.7	66.8	7.4	0.67	F	n.s.	4≤5	15.8 ± 1.4	Spain
	Classical	Childhood	100	686.0	81.5	62.4	7.3	0.62	M	n.s.	4≤5	16.1 ± 1.5	Spain
	Classical	Childhood	87	677.1	71.8	63.5	7.7	0.61	F	n.s.	5≤6	16.3 ± 2.0	Spain
	Classical	Childhood	99	632.2	63.9	59.9	7.5	0.68	M	n.s.	5≤6	16.3 ± 1.8	Spain
	Classical	Childhood	118	635.0	72.8	61.3	7.3	0.73	F	n.s.	6≤7	16.5 ± 2.2	Spain
	Classical	Childhood	111	595.9	72.3	56.4	7.8	0.75	M	n.s.	6≤7	16.4 ± 2.1	Spain
	Classical	Childhood	117	602.8	60.1	59.0	7.0	0.67	F	n.s.	7≤8	16.9 ± 2.5	Spain
	Classical	Childhood	120	550.5	61.2	54.6	6.0	0.62	M	n.s.	7≤8	16.9 ± 2.3	Spain

	Classical	Childhood	123	565.3	61.2	56.8	7.6	0.79	F	n.s.	8≤9	17.4 ± 2.4	Spain
	Classical	Childhood	124	532.0	60.0	53.7	5.5	0.71	M	n.s.	8≤9	17.5 ± 2.8	Spain
	Classical	Childhood	130	538.5	62.8	52.6	7.0	0.77	F	n.s.	9≤10	17.9 ± 2.9	Spain
	Classical	Childhood	150	510.5	52.9	51.3	6.1	0.64	M	n.s.	9≤10	17.9 ± 2.9	Spain
	Classical	Childhood	128	504.0	64.2	49.3	7.6	0.75	F	n.s.	10≤11	18.6 ± 3.0	Spain
	Classical	Childhood	163	492.3	51.9	49.70	5.7	0.70	M	n.s.	10≤11	18.4 ± 2.7	Spain
	Classical	Adolescence	149	473.4	64.0	45.8	6.4	0.81	F	n.s.	11≤12	18.9 ± 2.8	Spain
	Classical	Adolescence	159	454.0	53.7	46.8	6.4	0.65	M	n.s.	11≤12	19.5 ± 3.2	Spain
	Classical	Adolescence	301	434.8	46.9	43.0	5.2	0.67	F	n.s.	12≤13	20.4 ± 3.0	Spain
	Classical	Adolescence	161	432.2	59.0	44.0	6.1	0.79	M	n.s.	12≤13	19.5 ± 3.1	Spain
	Classical	Adolescence	179	421.2	44.7	42.8	5.4	0.67	F	n.s.	13≤14	21.1 ± 2.6	Spain
	Classical	Adolescence	156	389.2	64.6	39.9	5.9	0.82	M	n.s.	13≤14	19.9 ± 2.8	Spain
	Classical	Adolescence	173	347.8	47.6	37.0	4.9	0.69	M	n.s.	14≤15	20.6 ± 3.0	Spain
	Classical	Adolescence	191	324.1	41.3	36.6	4.6	0.66	M	n.s.	15≤16	21.8 ± 3.2	Spain
	Classical	Adolescence	717	416.7	43.9	44.2	5.6	0.67	F	n.s.	15≤19	21.7 ± 2.8	Spain
**Redondo-del-Río M.P., 2019** [74]	Classical	Infancy	154	874.8	110.7	93.4	29.7	0.3839	F/M	n.s.	1.1 ± 0.75 days	n.s.	Spain
	Classical	Infancy	79	833.6	97.5	91.3	34.7	0.3412	M	n.s.	1.1 ± 0.8 days	n.s.	Spain
	Classical	Infancy	75	918.2	107.7	95.6	23.2	0.4812	F	n.s.	1.2 ± 0.8 days	n.s.	Spain
**Gomes T.L.M., 2021** [75]	Classical	Infancy	87	792.0	99.5	67.6	8.5	0.60	F/M	Multiethnic	3.4	15.3 ± 1.1	Brazil
	Classical	Infancy	100	846.0	130.1	60.3	8.8	0.63	F/M	Multiethnic	1	16.5 ± 1.3	Brazil
	Classical	Infancy	118	815.0	114.3	64.5	8.9	0.65	F/M	Multiethnic	2	15.6 ± 1.2	Brazil
	Classical	Infancy	151	852.7	119.5	66.4	9.2	0.41	F	Multiethnic	2.47 ± 0.85	15.8 ± 1.4	Brazil
	Classical	Infancy	154	784.8	105.4	61.7	8.6	0.57	M	Multiethnic	2.47±0.85	15.8 ± 1.2	Brazil
**Núñez-Ramos R., 2024** [78]	Classical	Infancy	40	840.01	161.61	86.65	18.15	0.4227	M	Caucasian	39 + 1 weeks (±2.12)	n.s.	Spain
	Classical	Infancy	45	897.27	120.11	87.00	20.02	0.4013	F	Caucasian	39 + 1 weeks (±2.12)	n.s.	Spain
	Classical	Infancy	85	870.33	143.21	86.84	19.05	0.4145	F/M	Caucasian	39 + 1 weeks (±2.12)	n.s.	Spain

Table S6. Children-adolescents population: characteristics and values for tolerance ellipse construction. BIVA, bioelectrical impedance vector analysis; R/H, resistance-to-height ratio; Xc/H, reactance-to-height ratio; SD, standard deviation; BMI, body mass index; M, male; F, female; n.s., not specified in the article.
