# Supplementary material for: Reference Tolerance Ellipses in Bioelectrical Impedance Vector Analysis Across General, Pediatric, Pathological, and Athletic Populations: A Scoping Review
Source: J Funct Morphol Kinesiol. 2025 Oct 22;10(4):415. doi: 10.3390/jfmk10040415 (PMC12641658; doi:10.3390/jfmk10040415)
Supplement: Supplementary file 1 [file jfmk-10-00415-s001.zip › Supplementary Table S7.pdf]

Table S7. Pathological population: characteristics and values for tolerance ellipse construction.

AUTHOR, YEAR	BIVA	PATHOLOGY	SAMPLE SIZE	R/H Mean Ohm/m	R/H SD Ohm/m	XC/H Mean Ohm/m	XC/H SD Ohm/m	*r*	SEX	ETHNICITY	AGE range Years	BMI range Kg/m^2	COUNTRY
**Toso S., 2000** [41]	Classical	Cancer	30	314	61	24	7	0.25	M	Caucasian	64.0 ± 7.0	25.0 ± 3.1	Italy
	Classical	Cancer	33	302	49	25	6	0.14	M	Caucasian	67 ± 5.0	25.0 ± 3.5	Italy
**Piccoli A., 1998** [39]	Classical	Edema	25	238	48.2	13.9	4.5	0.36	M	Caucasian	50 ± 14	25.2 ± 3.4	Italy
	Classical	Edema	25	290.8	59.8	15.2	4.6	0.61	F	Caucasian	57 ± 17	25.6 ± 4.5	Italy
	Classical	Obesity I-III	169	234.6	28.6	25.3	4.9	0.52	M	Caucasian	40 ± 14	41.3 ± 7.1	Italy
	Classical	Obesity I-III	371	299	43.5	30.2	7.2	0.63	F	Caucasian	43 ± 14	42.8 ± 7.5	Italy
**Guglielmi F.W., 1999** [40]	Classical	Cirrhosis with ascites	24	366	51	29	6	0.2	F	n.s.	56 ± 11	24 ± 3	Italy
	Classical	Cirrhosis with ascites	33	301	58	27	9	0.5	M	n.s.	58 ± 10	24 ± 3	Italy
	Classical	Chronic hepatitis	272	289	35	33	5	0.4	M	n.s.	48 ± 12	25 ± 3	Italy
	Classical	Cirrhosis	144	290	42	30	6	0.4	M	n.s.	55 ± 10	25 ± 3	Italy
	Classical	Chronic hepatitis	166	366	44	38	7	0.3	F	n.s.	50 ± 11	26 ± 4	Italy
	Classical	Cirrhosis with ascites and edemas	32	248	47	17	5	0.6	M	n.s.	57 ± 13	27 ± 3	Italy
	Classical	Cirrhosis with ascites and edemas	23	313	80	23	8	0.8	F	n.s.	58 ± 13	27 ± 4	Italy
	Classical	Cirrhosis	116	361	50	34	7	0.4	F	n.s.	58 ± 8	27 ± 4	Italy
**Bogonez P., 2003** [29]	Classical	COPD	101	295.17	37.5	29.8	3.50	0.51	M	Caucasian	70	19-30	Spain
**Nescolarde L., 2004** [50]	Classical	Haemodialysis patients with hyperhydrated and malnutrition states	12	283.5	43.9	17.9	2.6	0.7	F	n.s.	18–70	22.2 ± 2.4	Cuba
	Classical	Haemodialysis patients with hyperhydrated and malnutrition states	16	244.8	45.5	17.7	4.8	0.8	M	n.s.	18–70	22.5 ± 4.9	Cuba
	Classical	Haemodialysis patients with oedema	28	289.1	40.7	26.6	6.5	0.9	M	n.s.	18–70	23.1 ± 3.9	Cuba
	Classical	Haemodialysis patients with oedema	18	355.5	74.9	32.8	9.1	0.9	F	n.s.	18–70	25.0 ± 7.8	Cuba
**Siváková D., 2013** [52]	Classical	Parkinson's disease	2	227.47	23.13	30.73	1.28	1.00	M	n.s.	40–49	n.s.	Slovakia

	Classical	Parkinson's disease	5	297.06	15.31	33.59	10.92	0.86	F	n.s.	50–59	n.s.	Slovakia
	Classical	Parkinson's disease	10	251.38	40.80	31.69	6.37	0.78	M	n.s.	50–59	n.s.	Slovakia
	Classical	Parkinson's disease	12	325.37	41.29	32.24	4.17	0.14	F	n.s.	60–69	n.s.	Slovakia
	Classical	Parkinson's disease	10	245.70	28.74	30.76	6.09	0.90	M	n.s.	60–69	n.s.	Slovakia
	Classical	Parkinson's disease	7	353.78	45.77	36.22	5.17	0.44	F	n.s.	70–79	n.s.	Slovakia
	Classical	Parkinson's disease	10	242.44	44.41	25.59	6.04	0.80	M	n.s.	70–79	n.s.	Slovakia
	Classical	Parkinson's disease	2	310.92	60.74	29.13	2.37	1.00	F	n.s.	80 >	n.s.	Slovakia
	Classical	Parkinson's disease	2	257.36	26.35	26.17	3.41	1.00	M	n.s.	80 >	n.s.	Slovakia
**Jiang FL., 2023** [70]	Classical	Sarcopenia	14	429.9	61.0	35.8	9.0	0.75	F	Asiatic	75.1 ± 4.4	22.9 ± 2.2	Korea
	Classical	Sarcopenia	10	312.1	25.8	29.1	5.8	0.71	M	Asiatic	76.6 ± 4.2	23.6 ± 2.3	Korea
**Lim S.-K., 2025** [79]	Classical	Hip fractures	24	337.9	67.6	24.4	8.0	0.405	M	Asiatic	78.5 ± 5.2	21.4 ± 4.4	Korea
	Classical	Hip fractures	103	386.8	75.3	25.7	7.7	0.461	F/M	Asiatic	81.7 ± 6.2	22.1 ± 3.7	Korea
	Classical	Hip fractures	79	401.7	71.4	26.1	7.6	0.393	F	Asiatic	82.7 ± 6.2	22.3 ± 3.5	Korea
**Piccoli A., 1998** [38]	Classical	Hemodialis patients with no hemodynamic instability	436	353.6	44.9	29.3	7.3	0.38	F	Caucasian	60 ± 14	23.5 ± 3.1	Italy
	Classical	Hemodialis patients with no hemodynamic instability	680	292.6	40.6	26.3	5.8	0.32	M	Caucasian	58 ± 14	23.6 ± 2.9	Italy
	Classical	Hemodialis patients with hemodynamic instability	118	302.0	46.0	23.8	8.0	0.31	M	Caucasian	63 ± 14	24.1 ± 2.9	Italy
	Classical	Hemodialis patients with hemodynamic instability	133	371.5	61.1	27.6	9.1	0.28	F	Caucasian	65 ± 14	24.3 ± 3.1	Italy
**Piccoli A., 2004** [53]	Classical	CAPD, edema	22	338.8	49.0	18.8	3.6	0.39	F	Caucasian	67 ± 13	24.0 ± 4.3	Italy
	Classical	CAPD, no edema	77	303.0	44.9	28.0	6.3	0.41	M	Caucasian	59 ± 14	24.2 ± 2.7	Italy
	Classical	CAPD, no edema	72	377.9	58.2	33.4	7.9	0.52	F	Caucasian	58 ± 13	24.4 ± 3.7	Italy

	Classical	CAPD, edema	29	265.9	56.6	17.0	3.0	0.10	M	Caucasian	64 ± 12	25.1 ± 3.2	Italy
**Rossini-Venturini A.C., 2022** [68]	Classical	Sarcopenia	6	413.5	49.9	35.3	14.1	0.944	F	n.s.	75.3 ± 5.6	22.8 ± 3.5	Brazil
	Classical	Sarcopenia	4	313.1	46.7	31.3	8.4	0.998	M	n.s.	76.8 ± 6.6	22.8 ± 4.5	Brazil
	Specific	Sarcopenia	6	422.6	110.9	35.3	15.9	0.742	F	n.s.	75.3 ± 5.6	22.8 ± 3.5	Brazil
	Specific	Sarcopenia	4	356.7	109.5	35.7	13.5	0.967	M	n.s.	76.8 ± 6.6	22.8 ± 4.5	Brazil

Table S7. Pathological population: characteristics and values for tolerance ellipse construction. BIVA, bioelectrical impedance vector analysis; R/H, resistance-to-height ratio; Xc/H, reactance-to-height ratio; SD, standard deviation; BMI, body mass index; M, male; F, female; n.s., not specified in the article; COPD, chronic obstructive pulmonary disease; CAPD, continuous ambulatory peritoneal dialysis.
