# Supplementary material for: Reference Tolerance Ellipses in Bioelectrical Impedance Vector Analysis Across General, Pediatric, Pathological, and Athletic Populations: A Scoping Review
Source: J Funct Morphol Kinesiol. 2025 Oct 22;10(4):415. doi: 10.3390/jfmk10040415 (PMC12641658; doi:10.3390/jfmk10040415)
Supplement: Supplementary file 1 [file jfmk-10-00415-s001.zip › Supplementary Table S8.pdf]

Table S8. Athletic population: characteristics and values for tolerance ellipse construction.

AUTHOR, YEAR	BIVA	DISCIPLINE	SAMPLE SIZE	R/H Mean Ohm/m	R/H SD Ohm/m	XC/H Mean Ohm/m	XC/H SD Ohm/m	*r*	SEX	ETHNICITY	AGE range Years	BMI range Kg/m²	COUNTRY
**Micheli M.L., 2014** [54]	Classical	Soccer players (medium to low level)	186	270.4	26.9	33.3	3.7	0.72	M	Caucasian	22.6 ± 5.3	22.8 ± 1.7	Italy
	Classical	Soccer players (medium level)	153	270.6	28.6	33.8	4.1	0.75	M	Caucasian	22.2 ± 5.0	22.9 ± 1.8	Italy
	Classical	Soccer players (high level)	202	260.5	19.8	33.7	3.6	0.75	M	Caucasian	24.5 ± 5.0	23.2 ± 1.4	Italy
	Classical	Soccer players (low level)	133	271.7	27.8	34.2	4.2	0.71	M	Caucasian	24.7 ± 5.0	23.2 ± 1.7	Italy
	Classical	Soccer players (all levels)	893	263.9	26.2	33.8	3.9	0.69	M	Caucasian	24.1 ± 5.1	23.3 ± 1.6	Italy
	Classical	Soccer players (élite level)	219	252.1	23.1	33.9	4.1	0.78	M	Caucasian	26.1 ± 4.4	23.9 ± 1.4	Italy
**Campa F., 2018** [56]	Classical	Volleyball (sub-élite group)	65	241.1	21.0	31.2	4.5	0.69	M	Caucasian	27.1 ± 6.1	23.3 ± 1.8	Italy
	Classical	Volleyball (low-level group)	61	242.3	23.7	32.9	3.2	0.71	M	Caucasian	24.8 ± 5.5	23.6 ± 2.4	Italy
	Classical	Volleyball, all group	201	238.6	4.3	31.8	4.2	0.7	M	Caucasian	26.1 ± 5.4	23.7 ± 2.0	Italy
	Classical	Volleyball (élite group)	75	232.1	24.1	31.5	4.3	0.72	M	Caucasian	26.3 ± 4.7	23.9 ± 1.8	Italy
	Classical	Road cyclist (professional climber group)	46	300.8	36.8	36.3	3.4	0.547	M	n.s.	26.2 ± 5.6	20.3 ± 1.2	Italy
**Giorgi A., 2018** [57]	Classical	Road cyclist (youth élite group)	59	264.1	40.7	33.7	3.7	0,57	M	n.s.	16.8 ± 1.1	20.9 ± 1.7	Italy
	Classical	Road cyclist (élite group)	79	284.5	31.4	34.9	4.1	0.568	M	n.s.	21.1 ± 2.9	21.8 ± 1.6	Italy
	Classical	Road cyclist (professional all-rounder group)	81	272.8	34.8	35.3	4.1	0.547	M	n.s.	26.5 ± 4.3	22.2 ± 1.3	Italy
	Classical	Road cyclist (professional sprinter group)	28	268.4	37.9	36.4	3.3	0.547	M	n.s.	26.1 ± 4.1	22.9 ± 1.3	Italy
	Classical	Road cyclist (amateurs group)	232	279.1	36.8	31.6	4.2	0.473	M	n.s.	39.0 ± 10.5	22.9 ± 2.8	Italy
**Koury J.C., 2018** [58]	Classical	Soccer players (late maturer)	12	438.0	55.0	43.7	5.3	0.762	M	n.s.	13.4 ± 0.6	16.6 ± 1.2	Brazil
	Classical	Soccer players (on time maturer)	18	321.6	37	37.2	3.8	0.620	M	n.s.	13.4 ± 0.6	18.7 ± 1.4	Brazil
	Classical	Soccer players (early maturer)	10	300.5	38.5	36.1	6.8	0.647	M	n.s.	13.4 ± 0.6	21.2 ± 2.2	Brazil
**Campa F., 2019** [28]	Classical	All athletes	440	318.1	42.8	38.3	6.4	0.70	F	n.s.	26.9 ± 6.6	21.9 ± 2.1	Italy

	Classical	Velocity/power[a]	177	321.0	46.9	38.0	7.4	0.80	F	n.s.	26.2 ± 5.5	22.1 ± 1.8	Italy
	Classical	Team sports[b]	187	305.6	37.6	36.3	5.3	0.60	F	n.s.	27.5 ± 7.4	22.1 ± 2.3	Italy
	Classical	Endurance[c]	76	337.5	42.9	40.1	5.5	0.60	F	n.s.	27.4 ± 7.0	21.4 ± 2.3	Italy
	Classical	All athletes	1116	251.6	32.5	33.9	4.8	0.70	M	n.s.	23.1 ± 6.8	23.7 ± 4.0	italy
	Classical	Velocity/power[a]	375	253.3	32.4	34.2	5.5	0.70	M	n.s.	23.6 ± 7.4	23.5 ± 2.5	Italy
	Classical	Team sports[b]	576	246.2	32.3	32.9	4.8	0.60	M	n.s.	22.7 ± 6.5	24.4 ± 4.4	Italy
	Classical	Endurance[c]	165	267.2	28.0	35.5	4.7	0.50	M	n.s.	23.5 ± 6.2	21.9 ± 4.1	Italy
**Toselli S., 2020** [59]	Classical	Soccer Players (youth élite group)	178	382.1	81.6	41.3	7.8	0.81	M	n.s.	12.1 ± 1.6	n.s.	Italy
**Marini E., 2020** [23]	Classical	Mixed[d]	63	331.5	41.2	39.6	6.4	0.729	F	Caucasian	20.7 ± 5.1	21.8 ± 2.1	Italy
	Classical	Mixed[d]	139	255.8	30.6	34.6	5.1	0.669	M	Caucasian	21.5 ± 5.0	22.9 ± 2.6	Italy
**Bongiovanni T., 2020** [61]	Classical	Soccer players (professional group)	131	246	32.1	34.3	5.1	0.70	M	n.s.	25.1 ± 4.7	23.5 ± 1.0	Italy
**Di Credico A., 2021** [62]	Classical	Handball	18	331.9	28.8	41.5	9.0	0.73	F	Caucasian	18.2 ± 0.7	23.0 ± 2.0	Italy
	Classical	Handball	37	258.0	25.0	34.9	4.0	0.69	M	Caucasian	17.5 ± 1.2	23.7 ± 2.5	Italy
**Abdelnour M., 2024** [76]	Classical	Endurance	29	414.6	36.6	39.9	3.9	0.39	F	n.s.	18-35	n.s.	United States
	Classical	Strenght	29	437.6	56.0	41.3	4.6	0.63	F	n.s.	18-35	n.s.	United States
	Classical	Endurance	36	303.7	32.0	35.0	4.9	0.47	M	n.s.	18-35	n.s.	United States
	Classical	Strenght	54	292.6	32.8	34.6	3.6	0.67	M	n.s.	18-35	n.s.	United States
**Cebrián-Ponce Á., 2024** [77]	Classical	Crossfit	38	338.4	39.9	39.3	5.2	0.67	F	Caucasian	28.1 ± 6.7	23.6 ± 2.8	Italy
	Classical	Crossfit	107	254.6	32.7	33.9	4.5	0.68	M	Caucasian	30.7 ± 8.4	26.0 ± 4.0	Italy
**Castizo-Olier J., 2018** [55]	Classical	Ultra-endurance triathletes	9	258.4	22.4	34.7	2.8	0.52	M	n.s.	36.6 ± 5.5	24.8 ± 2.0	Spain
**Nescolarde L., 2020** [60]	Classical	Marathon runners (non-élite group)	19	271.7	15.3	31.2	2.8	0.69	M	Caucasian	41.0 ± 4.0	24.0 ± 2.1	Spain
**Izzicupo P., 2023** [63]	Classical	Soccer referees (international èlite group)	43	276.3	39.5	37.4	5.1	0.7	M	Multiethnic	38.8 ± 3.6	23.2 ± 1.4	Italy
**Petri C., 2023** [64]	Classical	Body builders	41	221.1	26.4	31.2	4.0	0.81	M	White	30.1 ± 9.2	24.5 ± 1.5	Italy

			27	307.7	36.2	36.7	4.5	0.73	F	White	32.1 ± 8.0	19.8 ± 1.3	Italy
**Toselli S., 2020** [59]	Specific	Soccer Players (youth élite group)	178	300.9	35.9	32.8	5.1	0.64	M	n.s.	12.1 ± 1.6	n.s.	Italy
**Marini E., 2020** [23]	Specific	Mixed[d]	63	368.3	46.1	44.0	7.1	0.716	F	Caucasian	20.7 ± 5.1	21.8 ± 2.1	Italy
	Specific	Mixed[d]	139	324.3	31.2	43.9	6.2	0.636	M	Caucasian	21.5 ± 5.0	22.9 ± 2.6	Italy
**Cebrián-Ponce Á., 2024** [77]	Specific	Crossfit	38	355.8	60.7	41.2	6.4	0.81	F	Caucasian	28.1 ± 6.7	23.6 ± 2.8	Italy
	Specific	Crossfit	107	331.9	55.7	44.3	7.9	0.83	M	Caucasian	30.7 ± 8.4	26.0 ± 4.0	Italy

Table S8. Athletic population: characteristics and values for tolerance ellipse construction. BIVA, bioelectrical impedance vector analysis; R/H, resistance-to-height ratio; Xc/H, reactance-to-height ratio; SD, standard deviation; BMI, body mass index; M, male; F, female; n.s., not specified in the article;
a, Velocity/power: athletics, jumping, throwing, short-distance running, badminton, boxing, CrossFit, judo, karate, kickboxing, rhythmic gymnastics, short-distance swimming, and tennis;
b, Team sports: basketball, field hockey, handball, rugby, soccer, volleyball, and water polo;
c, Endurance: cycling, marathon, pentathlon, cross-country skiing, rowing, and triathlon;
d, Mixed: Athletics, Basketball, Handball, Judo, Karate, Pentathlon, Rugby, Soccer, Swimming, Triathlon, and Volleyball.
